# Synthesis and Evaluation of Thiazolidinone-Isatin Hybrids for Selective Inhibition of Cancer-Related Carbonic Anhydrases

**DOI:** 10.1021/acsmedchemlett.4c00599

**Published:** 2025-03-22

**Authors:** Alessia Onali, Erica Sanna, Antonio Lupia, Daniela Secci, Giulia Atzeni, Laura Demuru, Andrea Angeli, Filippo Cottiglia, Rita Meleddu, Roberta Emmolo, Angela Corona, Elias Maccioni, Claudiu T. Supuran, Simona Distinto

**Affiliations:** ‡ Department of Life and Environmental Sciences, 3111 University of Cagliari, Cittadella Universitaria sp8, 09042 Monserrato, Cagliari, Italy; † Faculty of Pharmacy, University of Lubiana, Aškerčeva cesta 7, 1000 Ljubljana, Slovenia; # Dipartimento NEUROFARBA, Sezione di Scienze Farmaceutiche, Università degli Studi di Firenze, 50019 Sesto Fiorentino, Florence, Italy

**Keywords:** Carbonic Anhydrases, CA inhibitors, Thiazolidinone-Isatin Hybrids, Docking Studies

## Abstract

A small library of novel thiazolidinone-based sulfonamide derivatives was designed, synthesized and evaluated for their ability to target human carbonic anhydrase (hCA) isoforms IX and XII, which are overexpressed in malignant cells and play a key role in metastasis and therapeutic response of cancer cells. A molecular hybridization approach was employed to design the molecules by combining different moieties identified as having antitumor activity. The thiazolidinone core was functionalized with benzenesulfonamide as a zinc-binding group and different isatin derivatives to enhance the chemical profile and optimize the hydrophilic/lipophilic balance. Biological evaluation against hCA I, II, IX and XII isoforms showed promising inhibitory activities, and some compounds exhibited selectivity and high inhibitory activity against hCA IX and hCA XII while not affecting off-target hCA I and hCA II. In particular, compound **3h** demonstrated high selectivity with K_i_ values of 57.8 nM for hCA IX and 44.3 nM for hCA XII.

## Graphical abstract

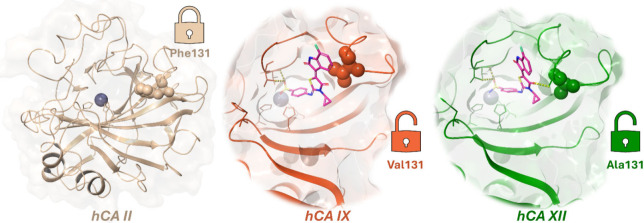

Carbonic anhydrases (CAs; also known as carbonate dehydratases, EC 4.2.1.1) are metalloenzymes that feature a metal ion at their active site, typically Zn^2+^ in α-CAs.^1^ Their primary function is to catalyze the reversible conversion of carbon dioxide and water into bicarbonate and protons, as shown in eq 1.^2^

1 $${\mathrm{CO}}_{2}+H_{2}O↔{{\mathrm{HCO}}_{3}}^{-}+H^{+}$$

In mammals, 16 different α-CA isoenzymes or CA-related proteins (CARPs) have been described, each with distinct subcellular localization and tissue-specific expression.^3^ These include several cytosolic forms (CA I, CA II, III, CA VII, CA XIII), five membrane-bound isozymes (CA IV, CA IX, CA XII, CA XIV and CA XV), a mitochondrial form (CA V), and a secreted (CA VI) found in saliva and milk.^4^ Additionally, three noncatalytic isoforms, known as CARPs VIII, X and XI, are predominantly expressed in the central nervous system.^5,6^ These enzymes are involved in a variety of physiological functions, including respiration, acid–base homeostasis, ion transport, bone resorption, and the secretion of gastric juice, aqueous humor and cerebrospinal fluid (CSF), depending on different CA isoenzymes.^7^ As a result, the identification of CA inhibitors (CAIs) represents an attractive strategy for the treatment of a wide range of conditions, including edema, glaucoma, obesity, cancer, epilepsy and osteoporosis.^8^

Although nonselective CA inhibition has been explored and found useful for therapeutic purposes, particularly in the treatment of glaucoma, epilepsy and high altitude sickness, such inhibitors often lack the specificity required to minimize off-target effects and adverse reactions, which can limit their clinical utility.^9^

Classical CAIs typically feature a zinc-binding group (ZBG), and the primary sulfonamides, such as acetazolamide and methazolamide, are well-known classical inhibitors.^10^ Although these inhibitors are highly potent and have been used in clinical applications for several decades, they lack isoform selectivity. Therefore, the aim of this work was to design and synthesize molecules that selectively target the isoforms involved in tumorigenesis.

CA IX is overexpressed in many tumors under hypoxic conditions, contributing to tumor acidosis by regulating pH and facilitating the development of metastatic traits.^11,12^ Similarly, CA XII is coexpressed with CA IX in various tumors and is highly expressed in cancers, including lung cancer and colorectal cancer.^13^ Both CA IX and CA XII are present in the cell membranes of tumor tissue, although their expression can vary, with some examples expressing only one or both isoforms.^14,12^

To selectively target hCA IX and XII, we employed a well-known drug design strategy known as the “tail approach”, linking chemical “tails” to aromatic sulfonamides. Thus, according to this information and following the previous studies conducted by our group^15–19^ we have further modified the scaffold.

Our strategy aims to enhance the interactions with amino acid residues located in the middle and at the edge of the active site cavity, where differences in amino acid sequences between the isoforms can be exploited.^10^

A molecular hybridization approach was applied to design the “tail” of these compounds,^20^ allowing us to combine two different fragments with known pharmaceutical properties to obtain thiazolidinone-isatin hybrids with potentially enhanced therapeutic activity. Thiazolidinones^21,22^ and isatins are considered privileged scaffolds in medicinal chemistry due to their biological activities and therapeutic potential across multiple disease targets. In particular, they have attracted considerable attention in the design of anticancer agents.^21,23,24^ The combination of these two chemical scaffolds aims to achieve a synergistic improvement in the pharmacological profile and the therapeutic potential of the synthesized compounds (Figure 1). To explore steric and conformational effects to increase affinity and selectivity toward biological targets, a cyclopropyl group was incorporated at position 3 of the thiazolidinone core. The cyclopropyl group was strategically used to increase potency, improve metabolic stability, and reduce off-target effects. Our group previously synthesized methyl substituted analogues^15^ and, according to tail approach, a bulkier substituent could improve the compounds selectivity. Furthermore, it provides a constraint in aliphatic systems while preserving a high sp^3^ ratio.^25^ It is present in numerous FDA-approved small molecule drugs, preclinical and clinical trials compounds, making it an attractive substituent in drug design.^26^

**Figure 1:**
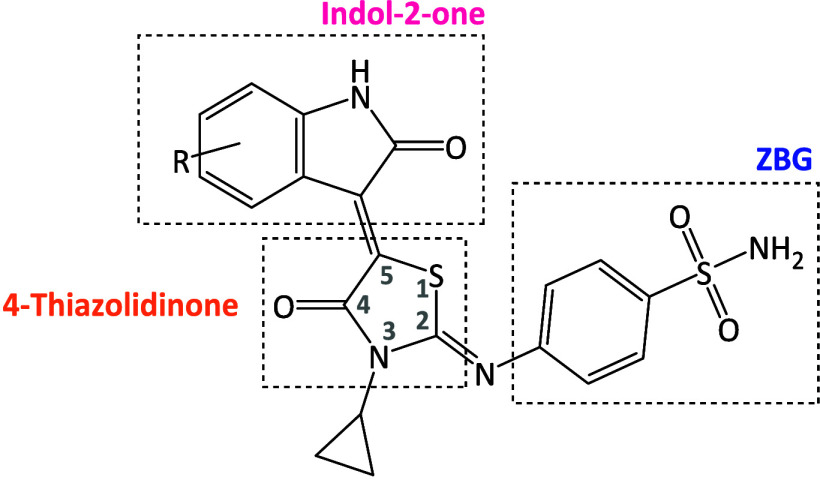
General structure of new thiazolidinone-isatin hybrids.

The final compounds **3a**–**i** were synthesized through a multistep reaction as described in Scheme 1.

**Scheme 1:**
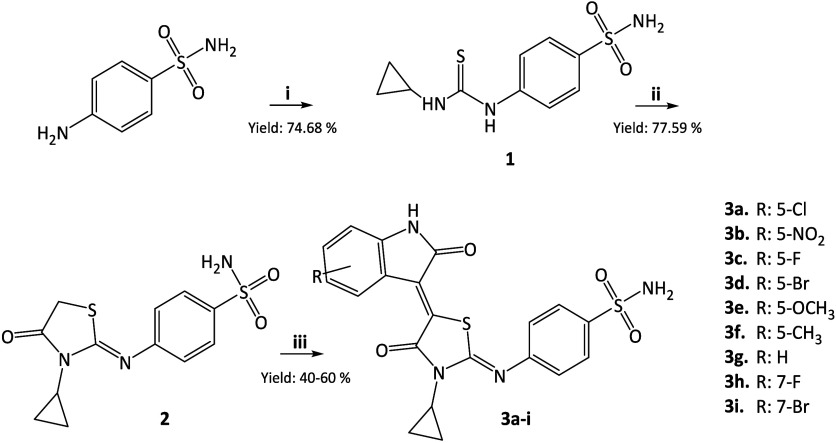
Reagents and Conditions: (i) Ethanol, Cyclopropyl Isothiocyanate; (ii) Ethanol, Ethyl Bromoacetate, Dry Sodium Acetate; and (iii) R-Isatin, Morpholine, Methanol

In the first step (i), 4-amino­benzen­sulfon­amide (1.00 g; 5.81 mmol) was refluxed in ethanol (15 mL) for 2–3 min. Cyclopropyl isothiocyanate (0.58 g; 5.81 mmol) was added dropwise and the mixture was refluxed for 48 h and then cooled to room temperature (rt). The resulting precipitate was filtered and crystallized from 2-propanol to obtain 4-(3-cyclo­propyl­thio­ureido) benzene­sulfon­amide **1** (1.18 g) as a white solid.

In the second step (ii), the thiazolidinone core was synthesized. An ethanol (20 mL) suspension of **1** (1.00 g; 3.69 mmol), and sodium acetate (1.81 g; 22.1 mmol) was stirred for 3 min. Subsequently, ethyl bromoacetate (0.49 mL; 4.42 mmol) was added, and the reaction mixture was refluxed under vigorous stirring for 48 h. Solvent was evaporated under reduced pressure. Ethyl acetate (50 mL) and 1 M HCl solution (20 mL) was added to the residue and the compound was extracted to the organic layer. The organic phase was dried over Na_2_SO_4_, filtered and the solvent removed under reduced pressure. To the crude product, ethanol was added, and the precipitate was filtered off to obtain **2** (0.89 g) as white solid.

In the final step (iii), the (Z)-4-((3-cyclo­propyl-4-oxothia­zolidin-2-ylid­ene)­amino)­benzene­sulfon­amide (**2**) was reacted overnight with the corresponding isatin in methanol and in the presence of morpholine as base under Knoevenagel condensation conditions. The crude solids were washed with ethanol/methanol to give derivatives **3a**–**i** in high purity. Detailed procedures can be found in the Supporting Information (SI), Section 1.2. In more detail, to diversify the chemical properties and optimize the hydrophilic/lipophilic balance, nine different isatin derivatives were incorporated at positions 5 and 7 of the central nucleus. All compounds were characterized using ^1^H and ^13^C nuclear magnetic resonance (NMR) spectroscopy, high-resolution mass spectrometry (HRMS), and X-ray crystallography. The purity has been assessed by HPLC and all compounds showed a purity of >95%. The ^1^H, ^13^C and HRMS spectra are reported in the SI (Figures S1–S23).

In hybrid thiazolidinone-isatin derivatives, two double bonds can lead to four isomeric configurations, ZZ, ZE, EE, and EZ. Crystallographic studies were carried out on the intermediate compound **2** to determine the configuration of the double bond at position 2 of the thiazolidinone ring. As compound **2** is a key intermediate, its configuration directly establishes the configuration of this bond in all the final compounds **3a**–**i**. These analyses confirmed the Z configuration (Figure 2A).

**Figure 2:**
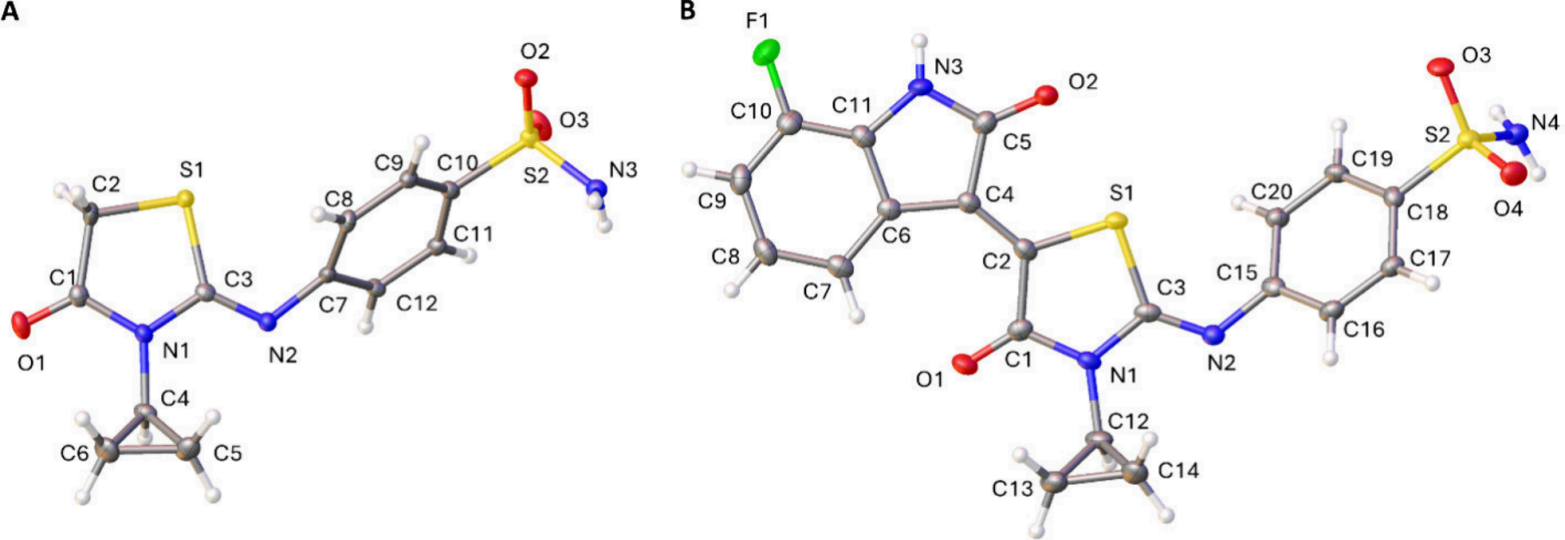
(A) X-ray structure of compound **2** in the Z configuration and (B) X-ray structure of compound **3h** in the ZZ configuration.

During the third step of the synthesis, a double bond was formed between position 5 of the thiazolidinone ring and position 3 of the indolinone moiety. According to the literature, only the Z isomer was formed.^27^ However, to further confirm the double bond geometry, crystallographic studies were performed on two final compounds, **3g** (reported in the SI) and **3h**. These analyses confirmed the ZZ configuration of the double bond at the position 5 of the thiazolidinone core. For reference, the crystallographic structure of compound **3h** is shown in Figure 2B.

The new derivatives were tested to prove their inhibitory activity and selectivity against hCA I, II, IX and XII isoforms. The CA catalyzed CO_2_ hydration/inhibition was measured using a stopped-flow instrument as previously described method.^28^ Acetazolamide (AAZ), a known potent but nonselective hCA inhibitor, was used as the reference standard. The biological evaluation results, summarized in Table 1, provide an overview of the inhibitory profiles of these novel derivatives against different hCA isoforms and highlight their potential for selective inhibition of the hCA IX and XII isoforms implicated in cancer. In particular, compound **3h**, which bears a fluorine atom at position 7 of the indol-2-one ring, stands out. The compound exhibits high selectivity for isoforms IX and XII (with Selectivity Index values of hCA II/hCA IX = 5.89 and hCA II/hCA XII = 7.69), with K_i_ values of 57.8 nM and 44.3 nM, respectively. The fluorine atom appears to confer an optimal balance between steric and electronic effects, thereby enhancing interactions with the active sites of these isoforms. In contrast, replacing the fluorine atom with a bromine atom (**3i**) leads to a complete loss of activity and selectivity for hCA IX. A possible explanation is that the presence of bromine increases steric hindrance, which may prevent the compound from interacting optimally with the active site of the enzyme. Furthermore, displacing the fluorine atom at position 5 of the indole-2-one ring, as shown in compound **3c**, resulted in a significantly reduced selectivity. The K_i_ values for hCA IX and XII are 88.1 nM and 73.0 nM, respectively, while the K_i_ for hCA II is 31.1 nM. This indicates that the fluorine positioning is critical for maintaining high selectivity and activity. Other compounds in the series also show interesting results. Compounds **3e** and **3f**, featuring electron-donating groups (EDGs) such as methyl or methoxy ones, exhibit marked selectivity for hCA XII, with Selectivity Index values of hCA II/hCA XII = 5.66 and hCA II/hCA XII = 4.38, respectively. There is a significant increase in activity when the methyl group (**3f**) is replaced by the isosteric chlorine atom (**3a**), which is more electronegative and thus has stronger electron-withdrawing (EWG) properties. This effect is particularly pronounced against hCA IX, where the K_i_ value improves dramatically from 551.5 nM to 49.2 nM. Meanwhile, increasing the electronegativity by introducing a nitro group at position 5, as in compound **3b**, increases the selectivity toward hCA II, with a K_i_ of 9.3 nM. This suggests its potential development for hCA II associated diseases such as diabetes. Conversely, compound **3g**, which maintains an unmodified isatin moiety, exhibits a K_i_ of 78.5 nM for hCA IX but lacked significant selectivity for this isoform. This highlights the need for further structural modifications to improve targeting. Furthermore, compound **2**, the thiazolidinone derivative applied as a synthon for condensation with isatin, does not show any selectivity toward isoforms IX and XII, highlighting that the central core must be functionalized to achieve isoform-specific activity. Compared to AAZ, with K_i_ values of 250.0 nM (hCA I), 12.1 nM (hCA II), 25.8 nM (hCA IX), and 5.7 nM (hCA XII), some compounds in this series demonstrate greater selectivity, particularly toward hCA IX and XII. This suggests that these novel derivatives have the potential to act as more selective inhibitors and deserve further development.

**Table 1:** Data Inhibition toward hCA I, II, IX and XII of **2** and **3a**–**i** Compounds Using AAZ as a Positive Control and Selectivity Index for hCA IX and XII as the Ratio of K_i_ Values over Off-Target hCA I and II

	K_i_ (nM)					selectivity index			
compound	R	hCA I	hCA II	hCA IX	hCA XII	hCA I/hCA IX	hCA I/hCA XII	hCA II/hCA IX	hCA II/hCA XII
**2**		86.2	15.9	74.8	71.8	1.15	1.20	0.21	0.22
**3a**	-5Cl	5134	60.9	49.2	140.7	104.35	36.49	1.24	0.43
**3b**	-5NO_2_	389.9	9.3	117.4	136.8	3.32	2.85	0.08	0.07
**3c**	-5F	3190	31.1	88.1	73.0	36.20	43.70	0.35	0.43
**3d**	-5Br	3866	199.3	89.6	240.2	43.15	16.09	2.22	0.83
**3e**	-5OCH_3_	5120	529.8	3358	93.6	1.52	54.70	0.16	5.66
**3f**	-5CH_3_	4174	362.1	551.5	82.6	7.57	50.53	0.66	4.38
**3g**	-H	950.5	76.7	78.5	195.1	12.11	4.87	0.98	0.39
**3h**	-7F	3465	340.6	57.8	44.3	59.95	78.22	5.89	7.69
**3i**	-7Br	4994	654.3	3511	93.5	1.42	53.41	0.19	7.00
AAZ		250.0	12.1	25.8	5.7	9.69	43.86	0.47	2.10

Molecular docking studies were carried out to elucidate the structure–activity relationship further, focusing on the most promising compound **3h**, predicting its binding interactions with hCA II, hCA IX and hCA XII. The crystal structures of hCA II, IX, and XII (hCAII: 3HS4,^29^ hCA IX: 3IAI,^30^ and hCA XII: 1JD0)^31^ were taken from the RCSB Protein Data Bank (PDB).^32^ Detailed procedures can be found in the SI.

Analysis of the amino acid sequences in the binding site reveals important differences across these isoforms. In hCA II, Phe at position 131 is replaced by Val in IX and Ala in XII. The bulky side chain at position 131 of hCA II acts as a “steric lock” in hCA II. This contrasts with the less bulky hydrophobic side chains in hCA IX and XII, which allow a more accessible active site. These structural differences may explain the different selectivity of the compounds between the isoforms.

To act as a zinc binder, the sulfonamide moiety should be located deeper in the active site allowing the inhibitor to establish the classical coordination cluster involving the zinc ion and the three histidine residues: His94, His96, and His119.

The results demonstrated that the compound **3h** is not able to coordinate the zinc ion in the catalytic sites of hCA II (Figure 3). Actually, it remains outside the active site, likely due to the steric hindrance of Phe131, which prevents compound **3h** from achieving an optimal interaction. This helps to clarify the reason for the low activity of this compound on this isoform. Compound **3h** only interacts with Arg58 at the gateway of the hCA II catalytic cavity.

**Figure 3:**
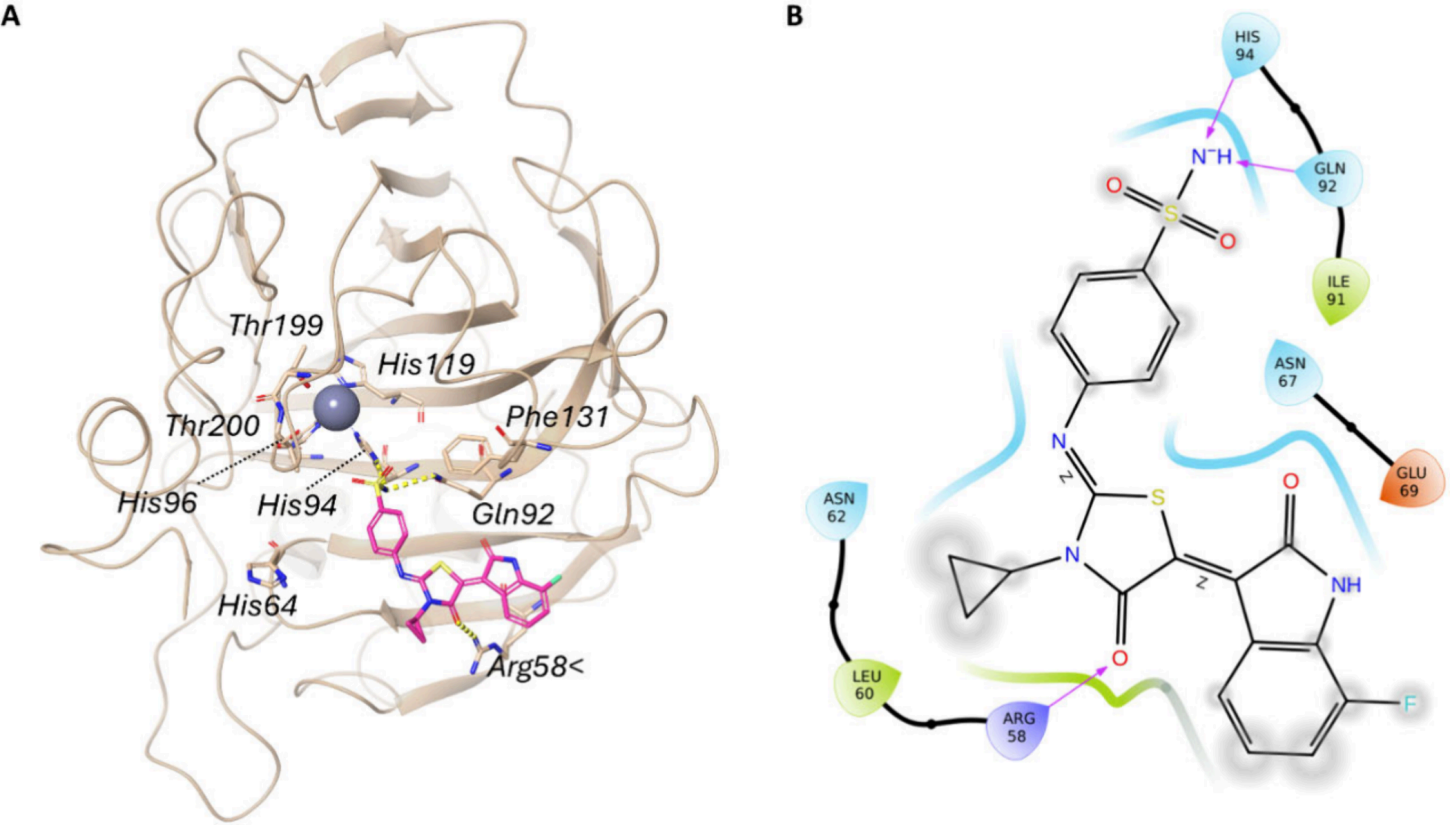
The putative binding mode of the most active compound (**3h**) in hCA II is shown. (A) 3D interactions between the ligand (magenta sticks) and the active site of hCA II (pale beige cartoon) (PDB: 3HS4). The interacting residues and the catalytic triad (His94, His96, and His119) are shown as pale beige sticks, hydrogen bonds as dotted yellow lines. (B) 2D representation of ligand-enzyme interactions. Residues labeled in light blue indicate polar groups, green signifies hydrophobic residues, red negatively charged residues, and purple positively charged residues. The gray-shaded areas indicate exposure to the solvent. Arrows denote hydrogen bonds.

In contrast, the predicted binding mode of **3h** with hCA IX and hCA XII shows a marked difference, as the compound effectively coordinates the zinc ion at the catalytic sites (Figures 4 and 5).

**Figure 4:**
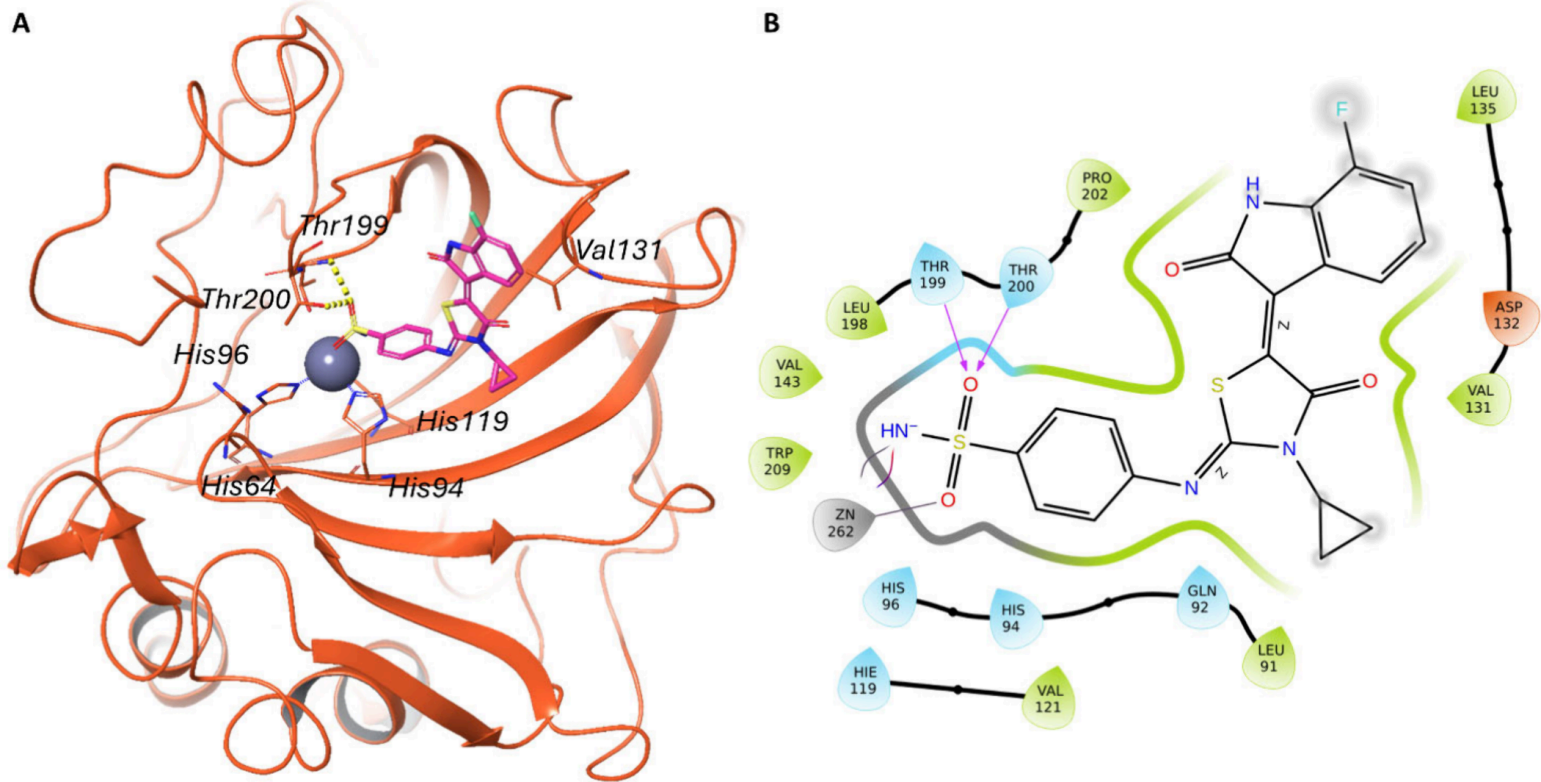
The putative binding mode of the most active compound (**3h**) in hCA IX is shown. (A) 3D interactions between the ligand (magenta sticks) and the active site of hCA IX (orange cartoon) (PDB: 3IAI). The interacting residues and the catalytic triad (His94, His96, and His119) are shown as orange sticks, hydrogen bonds as dotted yellow lines. (B) 2D representation of ligand-enzyme interactions. Residues labeled in light blue mark polar groups, green indicates hydrophobic residues, red negative charged residues, and purple positive charged residues. Hydrogen bonds are indicated by arrows, while metal coordination is indicated by a gray line. The gray-shaded areas indicate exposure to the solvent.

**Figure 5:**
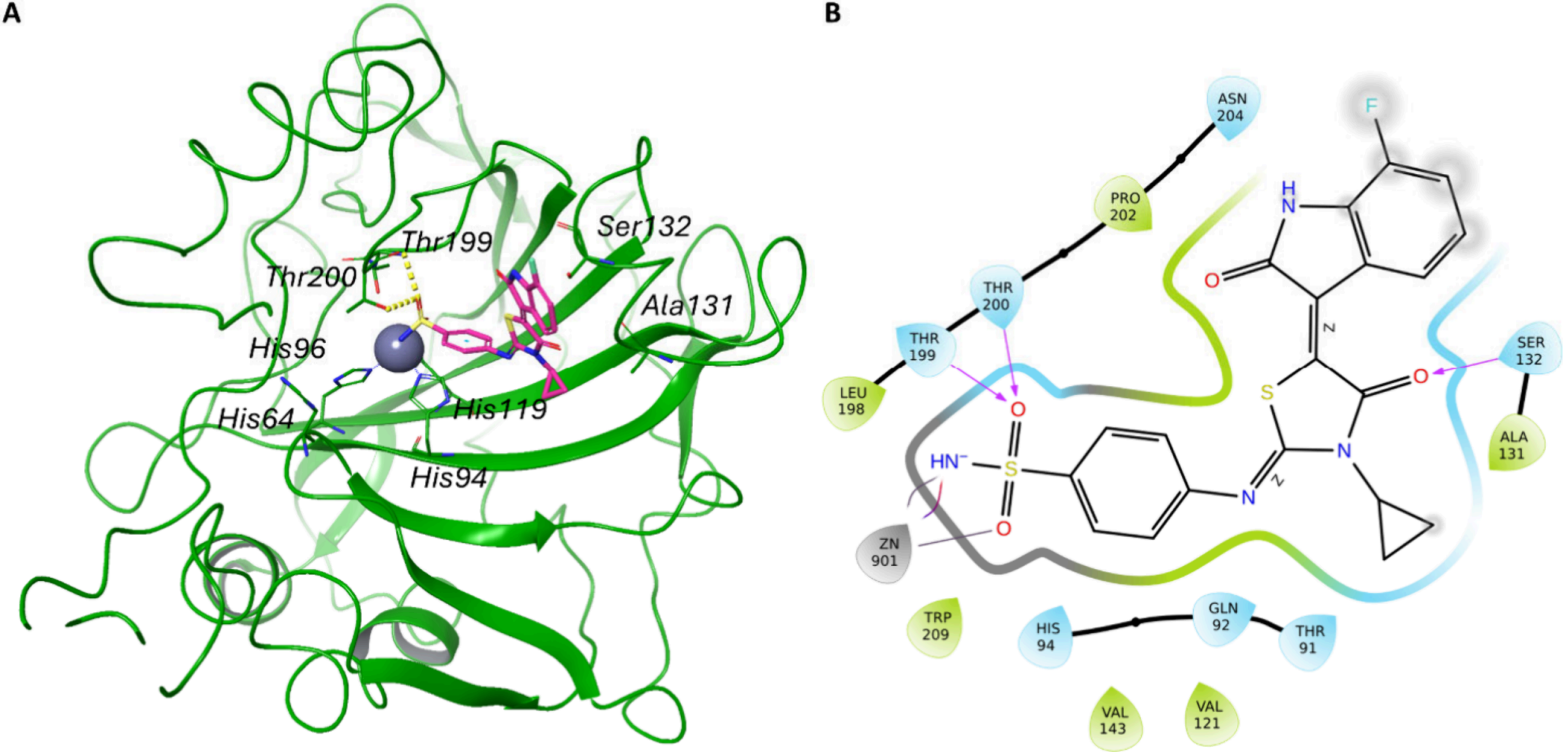
The putative binding mode of the most active compound (**3h**) in hCA XII is shown. (A) 3D interactions between the ligand (magenta sticks) and the active site of hCA XII (green cartoon) (PDB: 1JD0). The interacting residues and the catalytic triad (His94, His96, and His119) are shown as green sticks, hydrogen bonds as dotted yellow lines. (B) 2D representation of ligand-enzyme interactions. Residues labeled in light blue mark polar groups, green indicates hydrophobic residues, red negative charged residues, and purple positive charged residues. Arrows indicate hydrogen bonds, while a gray line indicates metal coordination. The gray-shaded areas indicate exposure to the solvent.

Of note, the **hCA IX-3h** complex is stabilized by the formation of hydrogen bonds with Thr199 and Thr200, as shown in Figure 4, panels A and B. Similarly, the **hCA XII-3h** complex maintains hydrogen bonds with Thr199 and Thr200, along with an additional bond to Ser132 (Figure 5). This consistent interaction with Thr199 and Thr200 in both isoforms IX and XII underlines the strong binding affinity of compound **3h** on these specific enzyme isoforms.

Structural modifications to further increase the activity and selectivity of these compounds are recommended. Moving forward, we plan to modify the substituent at position 3 of the thiazole ring, potentially improving interactions with isoforms IX and XII. Furthermore, as drug-like property prediction is crucial in early drug discovery for identifying and prioritizing promising compounds, the QikProp-predicted^33^ properties (Tables S3 and S4) indicate that most derivatives exhibit an interesting profile. In addition, the toxicity of compound **3h** was evaluated at 24, 48, and 72 h, and the results indicated that **3h** was nontoxic (Table S5). However, further structural scaffold optimization and experimental validation are needed to confirm the ADME predictions and assess their potential as valuable lead compounds.

In conclusion, while we have made significant progress toward improved activity against IX and XII isoforms, there is still room for optimization of activity, selectivity and pharmacokinetic properties.

## Supplementary Material



## Notes

Additional experimental details: materials, methods, synthesis, characterization data (^1^H and ^13^C NMR, MS spectra of all the newly synthesized compounds), X-ray crystallography, molecular modeling, theoretical prediction of drug-like properties, and biological assays (PDF)

## Abbreviations

hCA: human carbonic anhydrase
CSF: cerebrospinal fluid
CAIs: CA inhibitors
ZBG: zinc-binding group
NMR: nuclear magnetic resonance
HRMS: high-resolution mass spectrometry
PDB: Protein Data Bank
