# Supplementary material for: Synthesis and Evaluation of Thiazolidinone-Isatin Hybrids for Selective Inhibition of Cancer-Related Carbonic Anhydrases
Source: ACS Med Chem Lett. 2025 Mar 22;16(4):560–6. doi: 10.1021/acsmedchemlett.4c00599 (PMC11995222; doi:10.1021/acsmedchemlett.4c00599)
Supplement: Supplementary file 1 [file ml4c00599_si_001.pdf]

Supporting Information

# Synthesis and Evaluation of Thiazolidinone-Isatin Hybrids for Selective Inhibition of Cancer-Related Carbonic Anhydrases

*Alessia Onali[‡], Erica Sanna[‡*], Antonio Lupia[‡], Daniela Secci[†], Giulia Atzeni[‡], Laura Demuru[‡], Andrea Angeli[#], Filippo Cottiglia[‡], Rita Meleddu[‡], Roberta Emmolo[‡], Angela Corona[‡], Elias Maccioni[‡], Claudiu T. Supuran[#*] and Simona Distinto[‡].*

‡ Department of Life and Environmental Sciences, Cittadella Universitaria, University of Cagliari, sp8 09042 Monserrato, Cagliari, Italy

† Faculty of Pharmacy, University of Lubiana, Aškerčeva cesta 7, 1000 Ljubljana, Slovenia

# Dipartimento NEUROFARBA, Sezione di Scienze Farmaceutiche, Università degli Studi di Firenze, Sesto Fiorentino, Florence, Italy

# Table of contents

1. **Chemistry**
    - 1.1. Material and methods
    - 1.2. Synthesis and characterization
    - 1.3. $^{1}$H-NMR and $^{13}$C-NMR spectra (Figures S1-S11)
    - 1.4. Mass spectra of compounds (Figures S12-S22)
    - 1.5. X-Ray crystallography (Figure S23)

2. **Molecular modelling**
    - 2.1. Material and methods
    - 2.2. Qikprop prediction of drug-like properties

3. **Biological evaluation**
    - 3.1. Carbonic anhydrase inhibition assay
    - 3.2. Cellular toxicity assay

4. **References**

# 1. Chemistry

## 1.1. Material and methods

Reagents were obtained from commercial suppliers and used without any further purification. All reactions were monitored by thin layer chromatography (TLC) using precoated silica gel 60 F254 (Merck) with a layer thickness of 0.2 mm. The spots were visualised under UV detection (254 and 366 nm).

All melting points were determined by the capillary method on a Stuart Scientific melting point apparatus and are uncorrected.

Nuclear magnetic resonance (NMR) was recorded on a Bruker AMX 600 NMR spectrometer. $^{1}$H and $^{13}$C NMR spectra of samples were recorded at room temperature in 5 mm outside diameter (o.d.) tubes. Tetramethylsilane (TMS) was used as internal standard, chemical shifts (δ) are expressed in parts per million (ppm) and the coupling constant (J) in Hz. All samples were measured in DMSO-$d_6$ as a solvent.

Positive ESI-MS spectra were recorded with a high-resolution LTQ Orbitrap Elite™ mass spectrometer (Thermo Fisher Scientific). The solutions were infused at a flow rate of 5.00 μL/min into the ESI source. Spectra were recorded in the range of m/z 100-1500 with a resolution of 120000. The instrumental conditions were as follows. Spray voltage 3500 V, capillary temperature 275 °C, sheath gas 5-10 (arbitrary units), auxiliary gas 3 (arbitrary units), sweep gas 0 (arbitrary units), probe heater temperature 50 °C.

Single Crystal X-Ray diffraction data for compounds **2** and **3h** was collected at 100 K or 298 K on a Bruker D8 Venture diffractometer using a Mo Kα source and a PHOTON II area detector. The data were indexed and processed using Bruker SAINT [1] and SADABS [2]. The structures were solved with ShelXT [3] 2018/2 solution program using dual-space methods and refined with ShelXL [4] 2018/3 using full matrix least squares minimisation on F [2]. Olex2 [5] 1.5 was used as the graphical interface.

## 1.2. Synthesis and characterization

- *4-(3-cyclopropylthioureido)benzenesulphonamide* **(1)**

4-aminobenzensulphonamide (1.00 g; 5.81 mmol) was refluxed in ethanol (15 ml) for 2-3 minutes. Cyclopropyl isothiocyanate (0.58 g; 5.81 mmol) was added dropwise and the mixture was refluxed for 48 hours and then cooled to room temperature (rt). The resulting precipitate was filtered and

crystallised from 2-propanol to obtain **1** (1.18 g) as a white solid. The reaction was monitored by TLC (mobile phase: dichloromethane/methanol = 20:1) stained with ninhydrin.

Yield: 74.68%; White solid (1.18 g); mp: 215-217 °C; $R_f$ (dichloromethane/methanol = 20:1): 0.13. $^1$H NMR (600 MHz, DMSO-$d_6$) δ 0.52 – 0.69 (m, 2H, -C$\underline{H_2}$, cyclopropyl), 0.69 – 0.84 (m, 2H, -C$\underline{H_2}$, cyclopropyl), 2.79 – 3.08 (m, 1H, -C$\underline{H}$, cyclopropyl), 7.27 (s, 2H, -$SO_2N\underline{H_2}$), 7.59 – 7.82 (m, 4H, -C$\underline{H}$, 4-$SO_2NH_2$ phenyl), 8.21 (s, 1H, -NH, thiourea), 9.61 (s, 1H, -N$\underline{H}$, thiourea); $^{13}$C NMR (151 MHz, DMSO-$d_6$) δ 7.29, 26.81, 122.14, 126.47, 139.08, 143.37, 182.15.

m/z (ESI positive) theorical value for $C_{10}H_{13}N_3O_2S_2$: calculated 272.0522 found $[MH]^+$ 272.0535.

- *(Z)-4-((3-cyclopropyl-4-oxothiazolidin-2-ylidene)amino)benzenesulphonamide* **(2)**

An ethanol (20 ml) suspension of **1** (1.00 g; 3.69 mmol), and sodium acetate (1.81 g; 22.1 mmol) was stirred for 3 minutes. Subsequently, ethyl bromoacetate (0.49 ml; 4.42 mmol) was added, and the reaction mixture was refluxed under vigorous stirring for 48 hours. The solvent was evaporated under reduced pressure. Ethyl acetate (3 x 20 ml) and 1M HCl solution (20 ml) were added to the residue and the compound was extracted to the organic layer. The organic phase was dried over $Na_2SO_4$, filtered and the solvent removed under reduced pressure. To the crude product, ethanol was added, and the precipitate was filtered off to obtain **2** (0.89 g) as a white solid.

Yield: 77.59%; White solid (0.89 g); mp: 198-200 °C; $R_f$ (dichloromethane/methanol = 20:1): 0.24

$^1$H-NMR (600 MHz, DMSO-$d_6$) δ 0.89 – 1.01 (m, 4H, -C$\underline{H_2}$, cyclopropyl), 2.69 – 2.74 (m, 1H, -C$\underline{H}$, cyclopropyl), 3.96 (s, 2H, -C$\underline{H_2}$, tiazol.), 7.08 (d, 1H, $J$ = 8.6 Hz, -C$\underline{H}$, 4-$SO_2NH_2$ phenyl), 7.31 (s, 2H, -$SO_2N\underline{H_2}$), 7.81 (d, 1H, $J$ = 8.5 Hz, -C$\underline{H}$, 4-$SO_2NH_2$ phenyl); $^{13}$C NMR (151 MHz, DMSO-$d_6$) δ 6.90, 25.83, 32.87, 121.57, 127.61, 140.04, 152.29, 157.46, 172.56.

m/z (ESI positive) theorical value for $C_{12}H_{13}N_3O_3S_2$: calculated 312.0471, $[MH]^+$ found 312.0483.

- *4-(((Z)-5-((Z)-5-chloro-2-oxoindolin-3-ylidene)-3-cyclopropyl-4-oxothiazolidin-2-ylidene)amino)benzenesulphonamide* **(3a)**

To a suspension of **2** (0.200 g; 0.64 mmol) in methanol (10 ml); morpholine (0.07 ml; 0.77 mmol) was added dropwise. The reaction was stirred and after a few minutes a solution was obtained. 5-Chloroisatin (0.128 g; 0.71 mmol) was added and the reaction was stirred for 24 hours. HCl 37% (10 gtt) was added dropwise, and the reaction mixture was heated at 50 °C for 12 hours. The resulting precipitate was isolated by filtration. Subsequently, the residue was suspended in methanol, and after filtration, **3a** (0.183 g) was obtained as an orange solid.

Yield: 60.00 %; Orange solid (0.183 g); mp: > 300 °C; $R_f$ (dichloromethane/methanol = 20:1): 0.12

$^{1}H$ NMR (600 MHz, DMSO-$d_6$) δ 1.00 – 1.17 (m, 4H, -C<u>H</u>$_2$, cyclopropyl), 2.87 – 2.95 (m, 1H, -C<u>H</u>, cyclopropyl), 6.91 – 6.96 (m, 1H, *J* = 8.3 Hz, -C<u>H</u>, 5Cl isat.), 7.16 – 7.20 (m, 2H, -C<u>H</u>, 4-$SO_2NH_2$ phenyl), 7.36 (s, 2H, -$SO_2N$<u>H</u>$_2$), 7.42 (dd, 1H, *J* = 8.3, 2.0 Hz, -C<u>H</u>, 5Cl isat.), 8.81– 8.91 (m, 1H, -C<u>H</u>, 5Cl isat.), 7.78 – 7.95 (m, 2H, -C<u>H</u>, 4-$SO_2NH_2$ phenyl), 11.27 (s, 1H, -N<u>H</u>, 5Cl isatin); $^{13}C$ NMR (151 MHz, DMSO-$d_6$) δ 7.06, 25.81, 112.17, 121.71, 121.79, 123.86, 126.25, 127.61, 127.75, 131.57, 133.58, 140.66, 142.32, 151.40, 154.51, 166.53, 168.73.
m/z (ESI positive) theorical value for $C_{20}H_{15}ClN_4O_4S_2$: calculated 475.0296, $[MH]^+$ found 475.0326.

- *4-(((Z)-3-cyclopropyl-5-((Z)-5-nitro-2-oxoindolin-3-ylidene)-4-oxothiazolidin-2-ylidene)amino)benzenesulphonamide* **(3b)**

To a suspension of **2** (0.200 g; 0.64 mmol) in methanol (10 ml); morpholine (0.07 ml; 0.77 mmol) was added dropwise. Subsequently, 5-Nitroisatin was added, and the reaction mixture was stirred for 24 hours. 5-Nitroisatin (0.136 g; 0.71 mmol) was added and the reaction was stirred for 24 hours. HCl 37% (10 gtt) was added dropwise, and the reaction mixture was heated at 50 °C for 20 hours. The resulting precipitate was isolated by filtration, suspended in hot ethanol, filtered and the solid triturated in ethyl acetate. After the second filtration, compound **3b** (0.127 g) was obtained as an orange solid.
Yield: 40.71%; Orange solid (0.127 g); mp: > 300 °C; $R_f$ (dichloromethane/methanol = 20:1): 0.12
$^{1}H$ NMR (600 MHz, DMSO-$d_6$) δ 1.03 – 1.20 (m, 4H, -C<u>H</u>$_2$, cyclopropyl), 2.85 – 3.02 (m, 1H, -C<u>H</u>, cyclopropyl), 7.11 (d, 1H, *J* = 8.7 Hz, -C<u>H</u>, 5$NO_2$ isat.), 7.20 (d, 2H, *J* = 8.4 Hz, -C<u>H</u>, 4-$SO_2NH_2$ phenyl), 7.37 (s, 2H, -$SO_2N$<u>H</u>$_2$), 7.89 (d, 2H, *J* = 8.4 Hz, -C<u>H</u>, 4-$SO_2NH_2$ phenyl), 8.30 (dd, 1H, *J* = 8.4, 2.0 Hz, -C<u>H</u>, 5$NO_2$ isat.), 9.62 – 9.82 (m, 1H, -C<u>H</u>, 5$NO_2$ isat.), 11.87 (s, 1H, -N<u>H</u>, 5$NO_2$ isatin); $^{13}C$ NMR (151 MHz, DMSO-$d_6$) δ 7.09, 25.92, 111.03, 120.51, 121.71, 122.92, 123.56, 127.78, 128.05, 135.43, 140.79, 142.67, 148.79, 151.25, 154.08, 166.50, 169.36.
m/z (ESI positive) theorical value for $C_{20}H_{15}N_5O_6S_2$: calculated 486.0537, $[MH]^+$ found 486.0570.

- *4-(((Z)-3-cyclopropyl-5-((Z)-5-fluoro-2-oxoindolin-3-ylidene)-4-oxothiazolidin-2-ylidene)amino)benzenesulphonamide* **(3c)**

To a suspension of **2** (0.200 g; 0.64 mmol) in methanol (10 ml); morpholine (0.07 ml; 0.77 mmol) was added dropwise. The reaction was stirred and after a few minutes a solution was obtained. 5-Fluoroisatin (0.117 g; 0.71 mmol) was added and the reaction was stirred for 24 hours. HCl 37% (10 gtt) was added dropwise, and the reaction mixture was heated at 50 °C for 12 hours. The resulting

precipitate was isolated by filtration. Subsequently, the residue was suspended in ethanol, and after filtration, **3c** (0.127 g) was obtained as orange solid.
Yield: 43.20%; Orange solid (0.127 g); mp: > 300 °C; $R_f$ (dichloromethane/methanol = 20:1): 0.10
$^1$H NMR (600 MHz, DMSO-$d_6$) δ 1.01 – 1.18 (m, 4H, -C<u>H</u>$_2$, cyclopropyl), 2.81 – 2.99 (m, 1H, -C<u>H</u>, cyclopropyl), 6.92 (dd, 1H, *J* = 8.6, 4.7 Hz, -C<u>H</u>, 5F isat.), 7.16 – 7.20 (m, 2H, -C<u>H</u>, 4-$SO_2NH_2$ phenyl), 7.24 (td, 1H, *J* = 8.8, 2.8 Hz, -C<u>H</u>, 5F isat.), 7.35 (s, 2H, -$SO_2N$<u>H</u>$_2$), 7.85 – 7.91 (m, 2H, -C<u>H</u>, 4-$SO_2NH_2$ phenyl), 8.64 (dd, 1H, *J* = 10.3, 2.7 Hz, -C<u>H</u>, 5F isat.), 11.20 (s, 1H, -N<u>H</u>, 5F isatin); $^{13}$C NMR (151 MHz, DMSO-$d_6$) δ 7.04, 25.81, 111.54 (d, *J* = 8.4 Hz), 114.84, 115.02, 118.65 (d, *J* = 24.5 Hz), 121.23 (d, *J* = 10.2 Hz), 121.72, 124.52, 127.75, 133.35, 140.02, 140.64, 151.43, 154.59, 157.97 (d, *J* = 234.6 Hz), 166.58, 168.98.
m/z (ESI positive) theorical value for $C_{20}H_{15}FN_4O_4S_2$: calculated 459.0591, $[MH]^+$ found 459.0619.

- *4-(((Z)-5-((Z)-5-bromo-2-oxoindolin-3-ylidene)-3-cyclopropyl-4-oxothiazolidin-2-ylidene)amino)benzenesulphonamide* **(3d)**

To a suspension of **2** (0.200 g; 0.64 mmol) in methanol (10 ml); morpholine (0.07 ml; 0.77 mmol) was added dropwise. The reaction was stirred and after a few minutes a solution was obtained. 5-chloroisatin (0.160 g; 0.71 mmol) was added and the reaction was stirred for 24 hours. HCl 37% (15 gtt) was added dropwise, and the reaction mixture was heated at 50 °C for 12 hours. The precipitate was isolated by filtration, the residue was then suspended in ethanol and after a second filtration the compound **3d** (0.195 g) was obtained as a light brown solid.
Yield: 58.38%; Light brown solid (0.195 g); mp: > 300 °C; $R_f$ (dichloromethane/methanol = 20:1): 0.14
$^1$H NMR (600 MHz, DMSO-$d_6$) δ 1.03 – 1.16 (m, 4H, -C<u>H</u>$_2$, cyclopropyl), 2.81 – 2.96 (m, 1H, -C<u>H</u>, cyclopropyl), 6.89 (d, 1H, *J* = 8.3 Hz, -C<u>H</u>, 5Br isat.), 7.16 – 7.20 (m, 2H, -C<u>H</u>, 4-$SO_2NH_2$ phenyl), 7.35 (s, 2H, -$SO_2N$<u>H</u>$_2$), 7.54 (dd, 1H, *J* = 8.3, 2.1 Hz, -C<u>H</u>, 5Br isat.), 7.85 – 7.92 (m, 2H, -C<u>H</u>, 4-$SO_2NH_2$ phenyl), 9.01 (d, 1H, *J* = 2.0 Hz, -C<u>H</u>, 5Br isat.), 11.32 (s, 1H, -N<u>H</u>, 5Br isatin); $^{13}$C NMR (151 MHz, DMSO-$d_6$) δ 7.07, 25.81, 112.67, 113.99, 121.72, 122.28, 123.72, 127.75, 130.39, 133.61, 134.36, 140.66, 142.67, 151.40, 154.51, 166.56, 168.63.
m/z (ESI positive) theorical value for $C_{20}H_{15}BrN_4O_4S_2$: calculated 518.9791, $[MH]^+$ found 518.9817.

- *4-(((Z)-3-cyclopropyl-5-((Z)-5-methoxy-2-oxoindolin-3-ylidene)-4-oxothiazolidin-2-ylidene)amino)benzenesulphonamide* **(3e)**

To a suspension of **2** (0.200 g; 0.64 mmol) in methanol (10 ml); morpholine (0.07 ml; 0.77 mmol) was added dropwise. The reaction was stirred and after a few minutes a solution was obtained. 5-Methoxyisatin (0.125 g; 0.71 mmol) was added and the reaction was stirred for 24 hours. HCl 37% (20 gtt) was added dropwise, and the reaction mixture was heated at 50 °C for 28 hours. The resulting precipitate was isolated by filtration, the residue was then suspended in ethanol and after filtration **3e** (0.155 g) was obtained as an amber solid.

Yield: 51.32%; amber solid (0.155 g); mp: > 300 °C; $R_f$ (dichloromethane/methanol = 20:1): 0.10

$^1$H NMR (600 MHz, DMSO-$d_6$) δ 1.04 – 1.13 (m, 4H, -C<u>H</u>$_2$, cyclopropyl), 2.87 – 2.94 (m, 1H, -C<u>H</u>, cyclopropyl), 3.78 (s, 3H, -OC<u>H</u>$_3$, 5OCH$_3$ isat.), 6.83 (d, 1H, *J* = 8.5 Hz, -C<u>H</u>, 5OCH$_3$ isat.), 6.99 (dd, 1H, *J* = 8.5, 2.6 Hz, -C<u>H</u>, 5OCH$_3$ isat.), 7.18 (d, 2H, *J* = 8.4 Hz, -C<u>H</u>, 4-SO$_2$NH$_2$ phenyl), 7.35 (s, 2H, -SO$_2$N<u>H</u>$_2$), 7.88 (d, 2H, *J* = 8.4 Hz, -C<u>H</u>, 4-SO$_2$NH$_2$ phenyl), 8.54 (d, 1H, *J* = 2.5 Hz, -C<u>H</u>, 5OCH$_3$ isat.), 10.98 (s, 1H, -N<u>H</u>, 5OCH$_3$ isatin); $^{13}$C NMR (151 MHz, DMSO-$d_6$) δ 7.10, 25.76, 56.09, 111.09, 114.40, 117.89, 121.18, 121.74, 125.59, 127.73, 131.73, 137.48, 140.53, 151.56, 154.89, 155.06, 166.59, 168.97.

m/z (ESI positive) theorical value for $C_{21}H_{18}N_4O_5S_2$: calculated 471.0792, $[MH]^+$ found 471.0818.

- *4-(((Z)-3-cyclopropyl-5-((Z)-5-methyl-2-oxoindolin-3-ylidene)-4-oxothiazolidin-2-ylidene)amino)benzenesulphonamide* **(3f)**

To a suspension of **2** (0.200 g; 0.64 mmol) in methanol (10 ml); morpholine (0.07 ml; 0.77 mmol) was added dropwise. The reaction was stirred and after a few minutes a solution was obtained. 5-Methylisatin (0.114 g; 0.71 mmol) was added and the reaction was stirred for 24 hours. HCl 37% (10 gtt) was added dropwise, and the reaction mixture was heated at 50 °C for 12 hours. The resulting precipitate was isolated by filtration. Subsequently, the residue was suspended in methanol, and after filtration, **3f** (0.163 g) was obtained as a rust solid.

Yield: 55.82%; Rust solid (0.163 g); mp: > 300 °C; $R_f$ (dichloromethane/methanol = 20:1): 0.11

$^1$H NMR (600 MHz, DMSO-$d_6$) δ 1.01 – 1.17 (m, 4H, -C<u>H</u>$_2$, cyclopropyl), 2.33 (s, 3H, -C<u>H</u>$_3$, 5CH$_3$ isat.), 2.83 – 2.96 (m, 1H, -C<u>H</u>, cyclopropyl), 6.82 (d, 1H, *J* = 7.9 Hz, -C<u>H</u>, 5CH$_3$ isat.), 7.14 – 7.22 (m, 3H, C<u>H</u> + N<u>H</u>$_2$, 5CH$_3$ isat. + SO$_2$NH$_2$), 7.34 (s, 2H, -SO$_2$N<u>H</u>$_2$), 7.83 – 7.90 (m, 2H, -C<u>H</u>, 4-SO$_2$NH$_2$ phenyl), 8.70 (s, 1H, -C<u>H</u>, 5CH$_3$ isat.), 11.07 (s, 1H, -N<u>H</u>, 5CH$_3$ isatin); $^{13}$C NMR (151 MHz, DMSO-$d_6$) δ 7.11, 21.46, 25.71, 110.49, 120.03, 120.62, 121.74, 125.38, 127.72, 128.78, 131.12, 132.80, 140.51, 141.47, 151.61, 154.94, 166.55, 169.05.

m/z (ESI positive) theorical value for $C_{21}H_{18}N_4O_4S_2$: calculated 455.0842, $[MH]^+$ found 455.0870.

- *4-(((Z)-3-cyclopropyl-4-oxo-5-((Z)-2-oxoindolin-3-ylidene)thiazolidin-2-ylidene)amino)benzenesulphonamide* **(3g)**

To a suspension of **2** (0.200 g; 0.64 mmol) in methanol (10 ml); morpholine (0.07 ml; 0.77 mmol) was added dropwise. The reaction was stirred and after a few minutes a solution was obtained. 5-Isatin (0.104 g; 0.71 mmol) was added and the reaction was stirred for 24 hours. HCl 37% (15 gtt) was added dropwise, and the reaction mixture was stirred at for 20 hours. The resulting precipitate was isolated by filtration. Subsequently, the residue was suspended in methanol, and after filtration, **3g** (0.163 g) was obtained as an orange solid.

Yield: 59.27%; Orange solid (0.163 g); mp: > 300 °C; $R_f$ (dichloromethane/methanol = 20:1): 0.10
$^1$H NMR (600 MHz, DMSO-$d_6$) δ 1.02 – 1.12 (m, 4H, -C<u>H</u>$_2$, cyclopropyl), 2.86 – 2.96 (m, 1H, -C<u>H</u>, cyclopropyl), 6.93 (d, 1H, *J* = 7.7 Hz, -C<u>H</u>, isat.), 7.09 (t, 1H, *J* = 7.8 Hz, -C<u>H</u>, isat.), 7.15 – 7.20 (m, 2H, -C<u>H</u>, 4-$SO_2NH_2$ phenyl), 7.32 – 7.40 (m, 3H, C<u>H</u> + N<u>H</u>$_2$, isat. + $SO_2NH_2$), 7.85 – 7.90 (m, 2H, -C<u>H</u>, 4-$SO_2NH_2$ phenyl), 8.85 (d, *J* = 7.9 Hz, 1H, -C<u>H</u>, isat.), δ 11.17 (s, 1H, -N<u>H</u>, isatin); $^{13}$C NMR (151 MHz, DMSO-$d_6$) δ 7.05, 25.75, 110.78, 120.55, 121.74, 122.43, 125.16, 127.73, 128.41, 131.47, 132.35, 140.54, 143.67, 151.58, 154.88, 166.53, 169.01.
m/z (ESI positive) theorical value for $C_{20}H_{16}N_4O_4S_2$: calculated 441,0686, $[MH]^+$ found 441.0716.

- *4-(((Z)-3-cyclopropyl-5-((Z)-7-fluoro-2-oxoindolin-3-ylidene)-4-oxothiazolidin-2-ylidene)amino)benzenesulphonamide* **(3h)**

To a suspension of **2** (0.200 g; 0.64 mmol) in methanol (10 ml); morpholine (0.07 ml; 0.77 mmol) was added dropwise. The reaction was stirred and after a few minutes a solution was obtained. 7-Fluoroisatin (0.117 g; 0.71 mmol) was added and the reaction was stirred for 24 hours. HCl 37% (10 gtt) was added dropwise, and the reaction mixture was heated at 50 °C for 12 hours. The resulting precipitate was isolated by filtration. Subsequently, the residue was suspended in ethanol, and after filtration, **3h** (0.150 g) was obtained as an orange solid.

Yield: 51.02%; Orange solid (0.150 g); mp: > 300 °C; $R_f$ (dichloromethane/methanol = 20:1): 0.17
$^1$H NMR (600 MHz, DMSO-$d_6$) 0.90 – 1.31 (m, 4H, -C<u>H</u>$_2$, cyclopropyl), 2.69 – 3.02 (m, 1H, -C<u>H</u>, cyclopropyl), 7.10 (td, 1H, *J* = 8.2, 5.2 Hz, -C<u>H</u>, 7F isat.), 7.16 – 7.21 (m, 2H, -C<u>H</u>, 4-$SO_2NH_2$ phenyl), 7.29 – 7.34 (m, 1H, -C<u>H</u>, 7F isat.), 7.36 (s, 2H, -$SO_2N$<u>H</u>$_2$), 7.82 – 7.93 (m, 2H, -C<u>H</u>, 4-$SO_2NH_2$ phenyl), 8.71 (d, 1H, *J* = 7.9 Hz, -C<u>H</u>, 7F isat.), 11.71 (s, 1H, -N<u>H</u>, 7F isatin); $^{13}$C NMR (151 MHz, DMSO-$d_6$) δ 7.04, 25.81, 118.76 (d, *J* = 16.9 Hz), 121.74, 122.93 (d, *J* = 5.4 Hz), 123.24 (d, *J* = 4.2 Hz), 124.23 (d, *J* = 3.9 Hz), 124.40 (d, *J* = 1.6 Hz), 127.75, 130.68 (d, *J* = 13.1 Hz), 133.59, 140.63, 147.06 (d, *J* = 242.2 Hz), 151.44, 154.49, 166.34, 168.84.

m/z (ESI positive) theorical value for $C_{20}H_{15}FN_4O_4S_2$: calculated 459.0592, $[MH]^+$ found 459.0628.

- *4-(((Z)-5-((Z)-7-bromo-2-oxoindolin-3-ylidene)-3-cyclopropyl-4-oxothiazolidin-2-ylidene)amino)benzenesulphonamide* **(3i)**

To a suspension of **2** (0.200 g; 0.64 mmol) in methanol (10 ml); morpholine (0.07 ml; 0.77 mmol) was added dropwise. Subsequently, 7-Bromoisatin (0.160 g; 0.71 mmol) was added and the reaction was stirred for 24 hours. HCl 37% (10 gtt) was added dropwise, and the reaction mixture was heated at 50 °C for 12 hours. The resulting precipitate was isolated by filtration. Subsequently, the residue was suspended in methanol, and after filtration, **3i** (0.187 g) was obtained as sandstone solid.

Yield: 55.99%; Sandstone solid (0.187 g); mp: > 300 °C; $R_f$ (dichloromethane/methanol = 20:1): 0.16

$^1$H NMR (600 MHz, DMSO-$d_6$) δ 1.05 – 1.13 (m, 4H, -C$\underline{H}_2$, cyclopropyl), 2.88 – 2.94 (m, 1H, -C$\underline{H}$, cyclopropyl), 7.05 (t, 1H, $J$ = 8.0 Hz, -C$\underline{H}$, 7Br isat.), 7.17 – 7.20 (m, 2H, -C$\underline{H}$, 4-$SO_2NH_2$ phenyl), 7.36 (s, 2H, -$SO_2N\underline{H}_2$), 7.57 (d, 1H, $J$ = 8.1 Hz, -C$\underline{H}$, 7Br isat.), 7.84 – 7.91 (m, 2H, -C$\underline{H}$, 4-$SO_2NH_2$ phenyl), 8.84 – 8.90 (m, 1H, -C$\underline{H}$, 7Br isat.), 11.36 (s, 1H, -N$\underline{H}$, 7Br isatin); $^{13}$C NMR (151 MHz, DMSO-$d_6$) δ 7.03, 25.81, 103.13, 121.74, 122.24, 123.88, 124.52, 127.27, 127.76, 133.73, 134.66, 140.63, 142.58, 151.44, 154.51, 166.33, 168.90.

m/z (ESI positive) theorical value for $C_{20}H_{15}BrN_4O_4S_2$: calculated 518,9791, $[MH]^+$ found 518.9817.

## 1.3. $^{1}$H-NMR and $^{13}$C-NMR spectra

**Figure S1.** $^{1}$H and $^{13}$C NMR spectrum of 4-(3-cyclopropylthioureido)benzenesulfonamide **(1)**

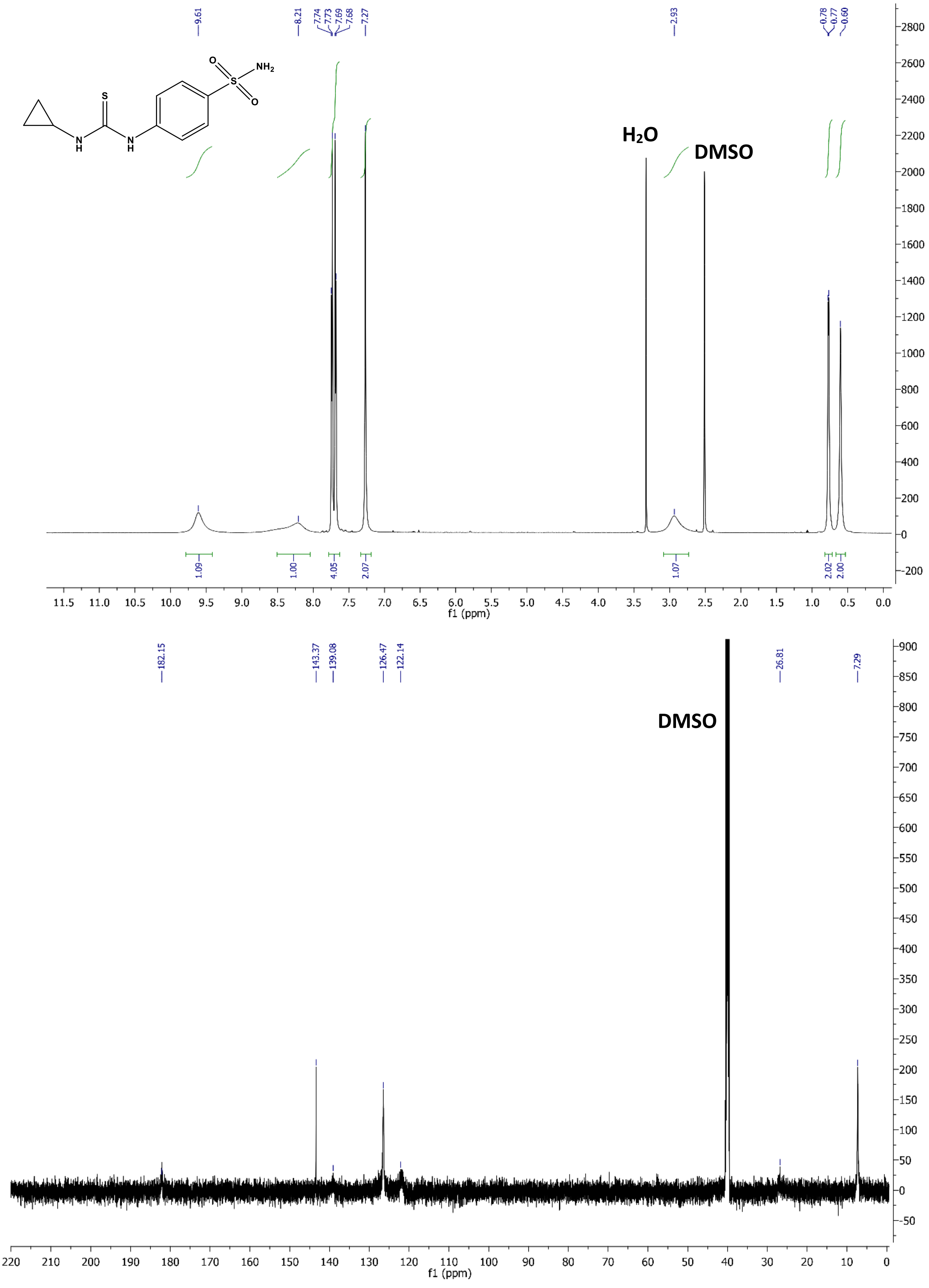

**Figure S2.** $^{1}H$ and $^{13}C$ NMR spectrum of (Z)-4-((3-cyclopropyl-4-oxothiazolidin-2-ylidene) amino)benzenesulfonamide **(2)**

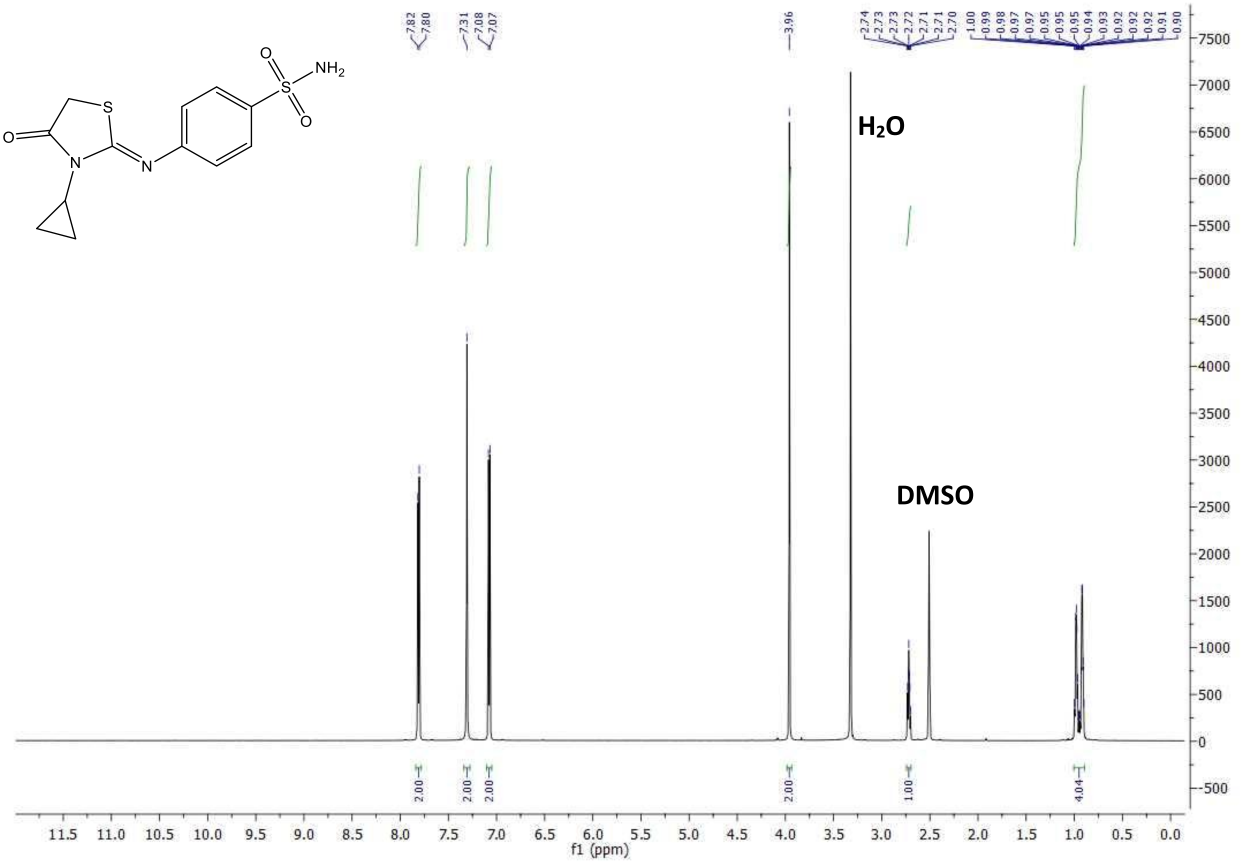


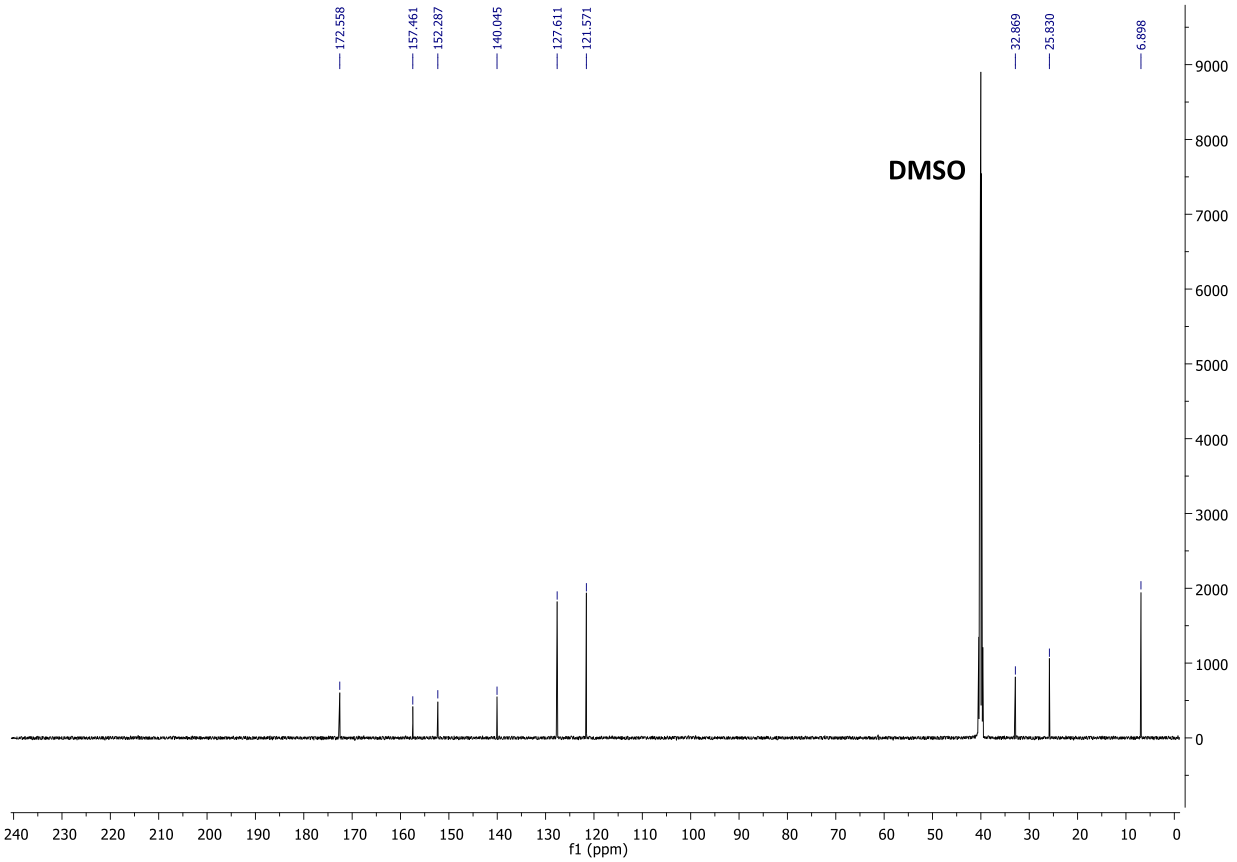

**Figure S3.** $^{1}$H and $^{13}$C NMR spectrum of 4-(((Z)-5-((Z)-5-chloro-2-oxoindolin-3-ylidene)-3-cyclopropyl-4-oxothiazolidin-2-ylidene)amino)benzenesulfonamide **(3a)**

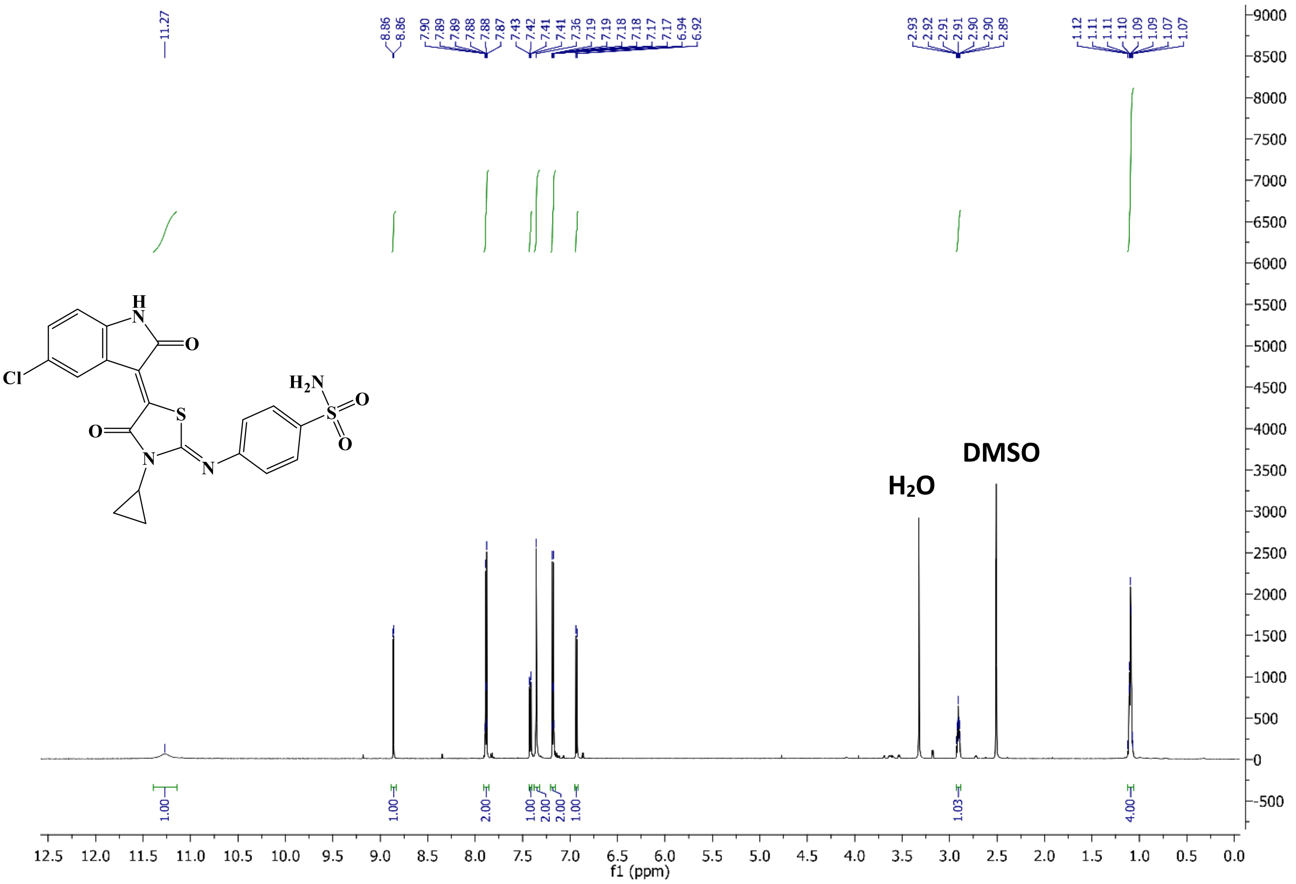


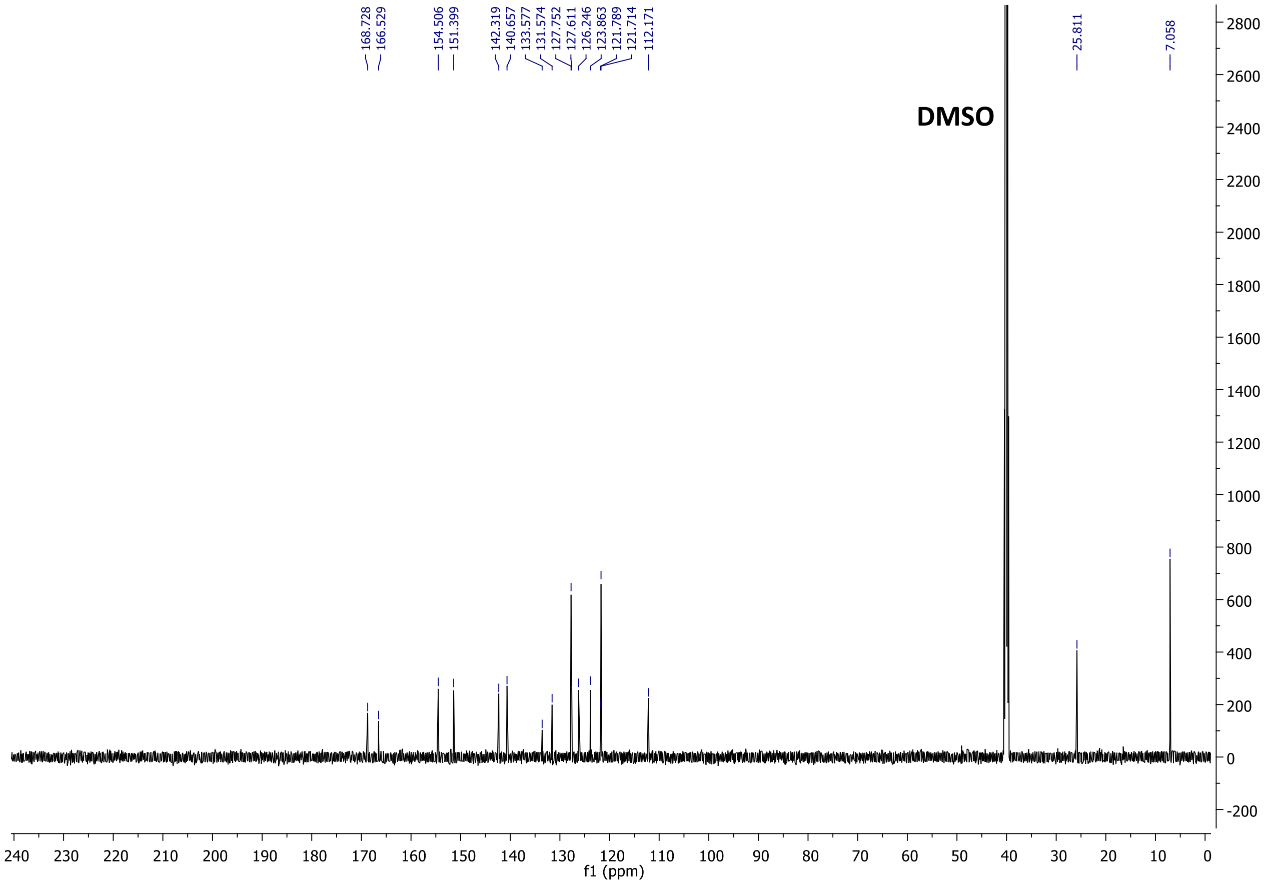

**Figure S4.** $^{1}$H and $^{13}$C NMR spectrum of 4-(((Z)-3-cyclopropyl-5-((Z)-5-nitro-2-oxoindolin-3-ylidene)-4-oxothiazolidin-2-ylidene)amino)benzenesulfonamide **(3b)**

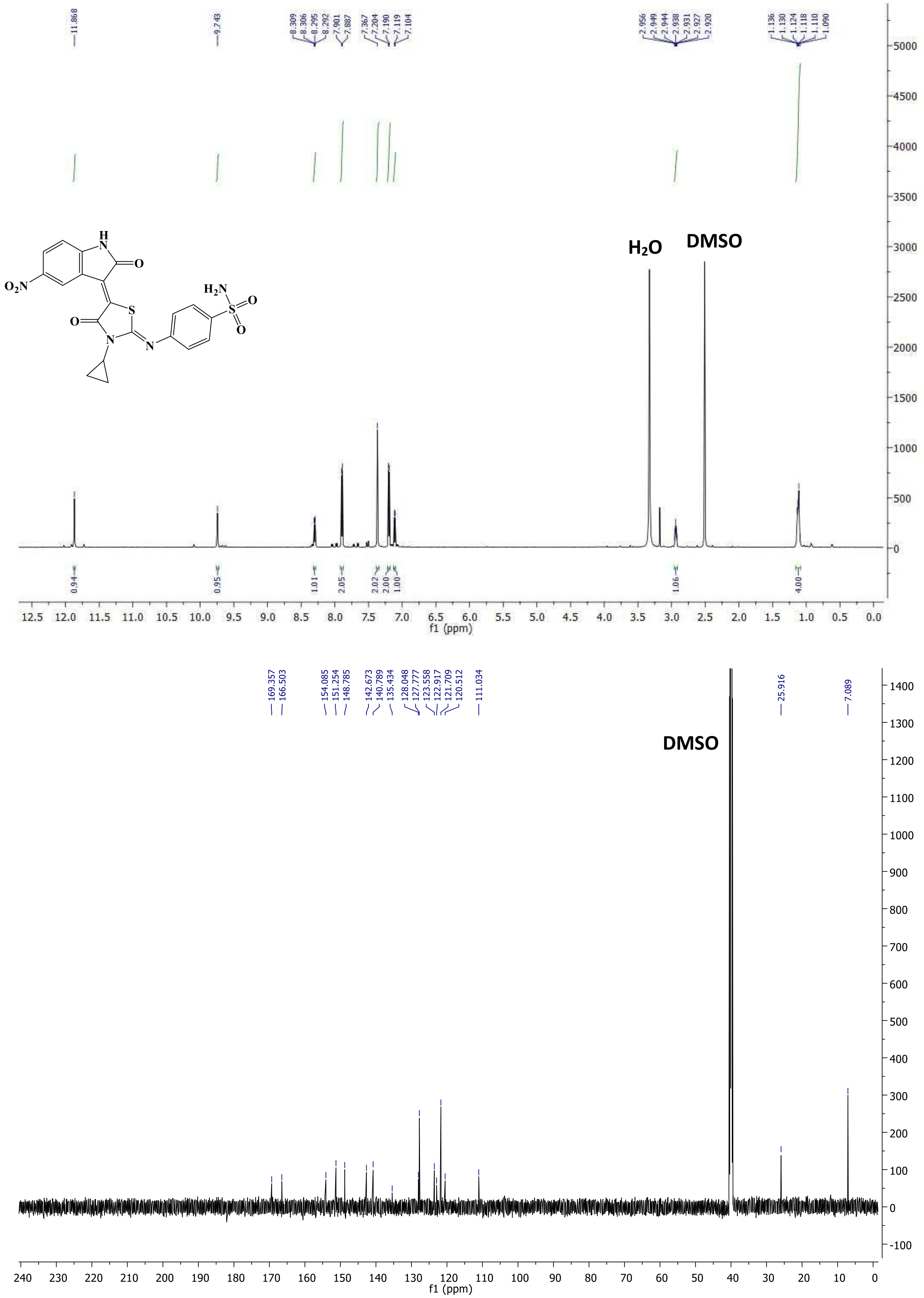

**Figure S5.** $^{1}H$ and $^{13}C$ NMR spectrum of 4-(((Z)-3-cyclopropyl-5-((Z)-5-fluoro-2-oxoindolin-3-ylidene)-4-oxothiazolidin-2-ylidene)amino)benzenesulfonamide **(3c)**

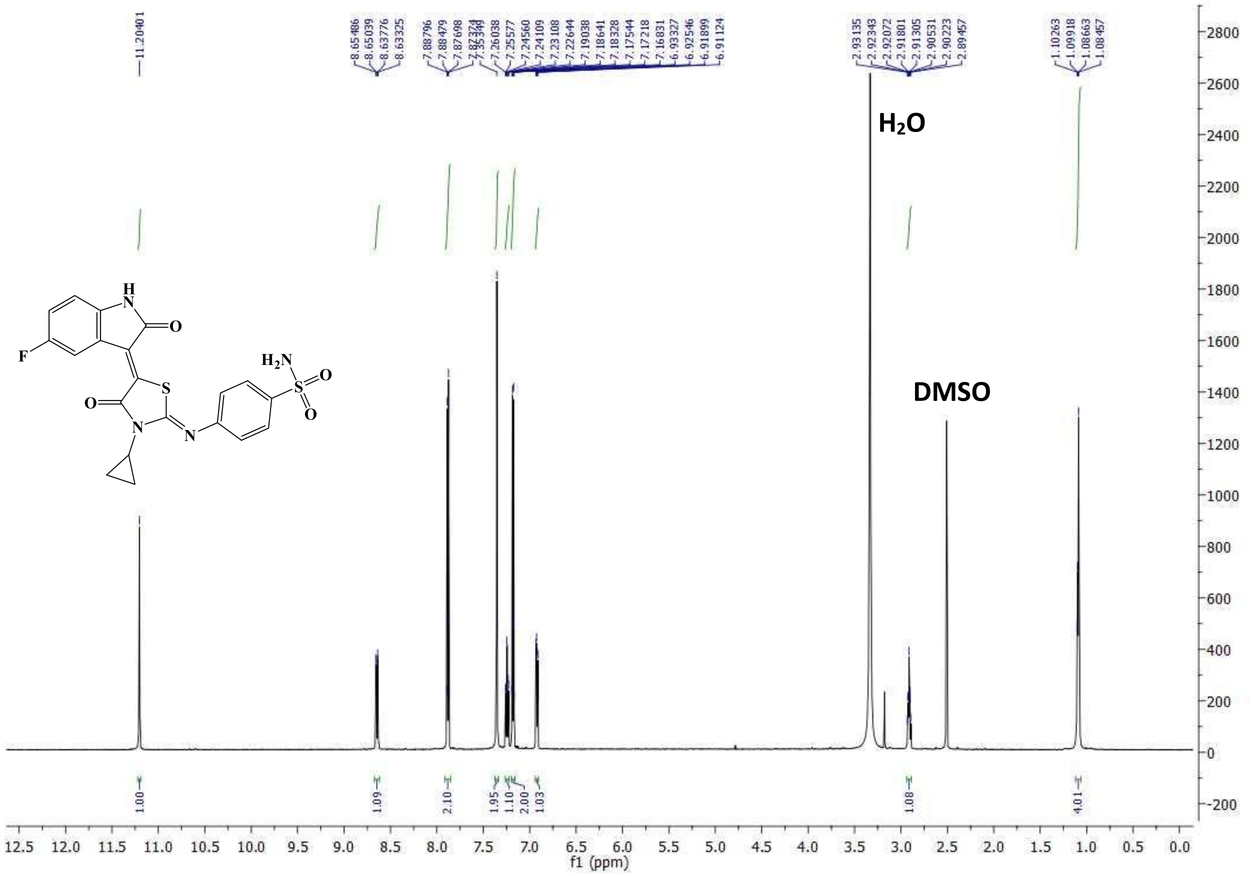


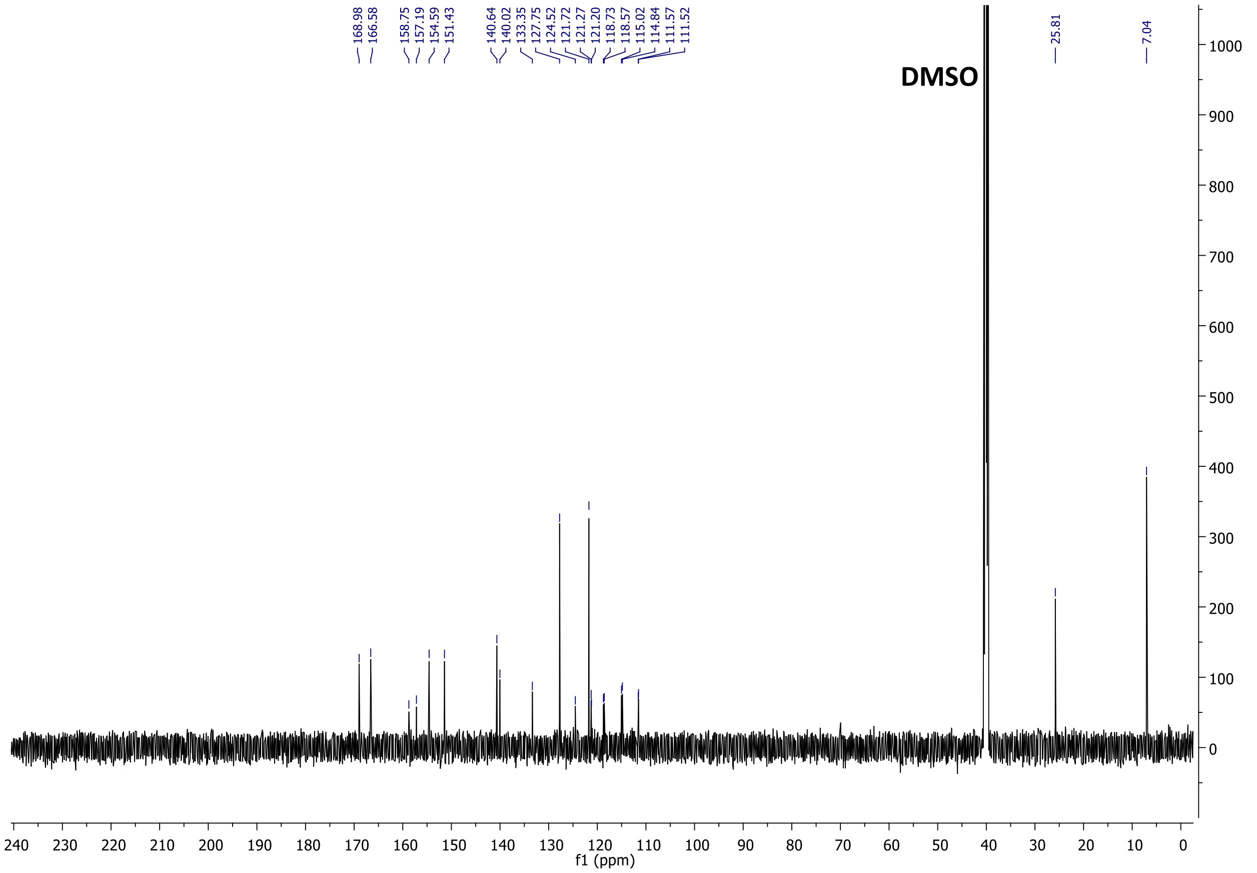

**Figure S6.** $^{1}$H and $^{13}$C NMR spectrum of 4-(((Z)-5-((Z)-5-bromo-2-oxoindolin-3-ylidene)-3-cyclopropyl-4-oxothiazolidin-2-ylidene)amino)benzenesulfonamide **(3d)**

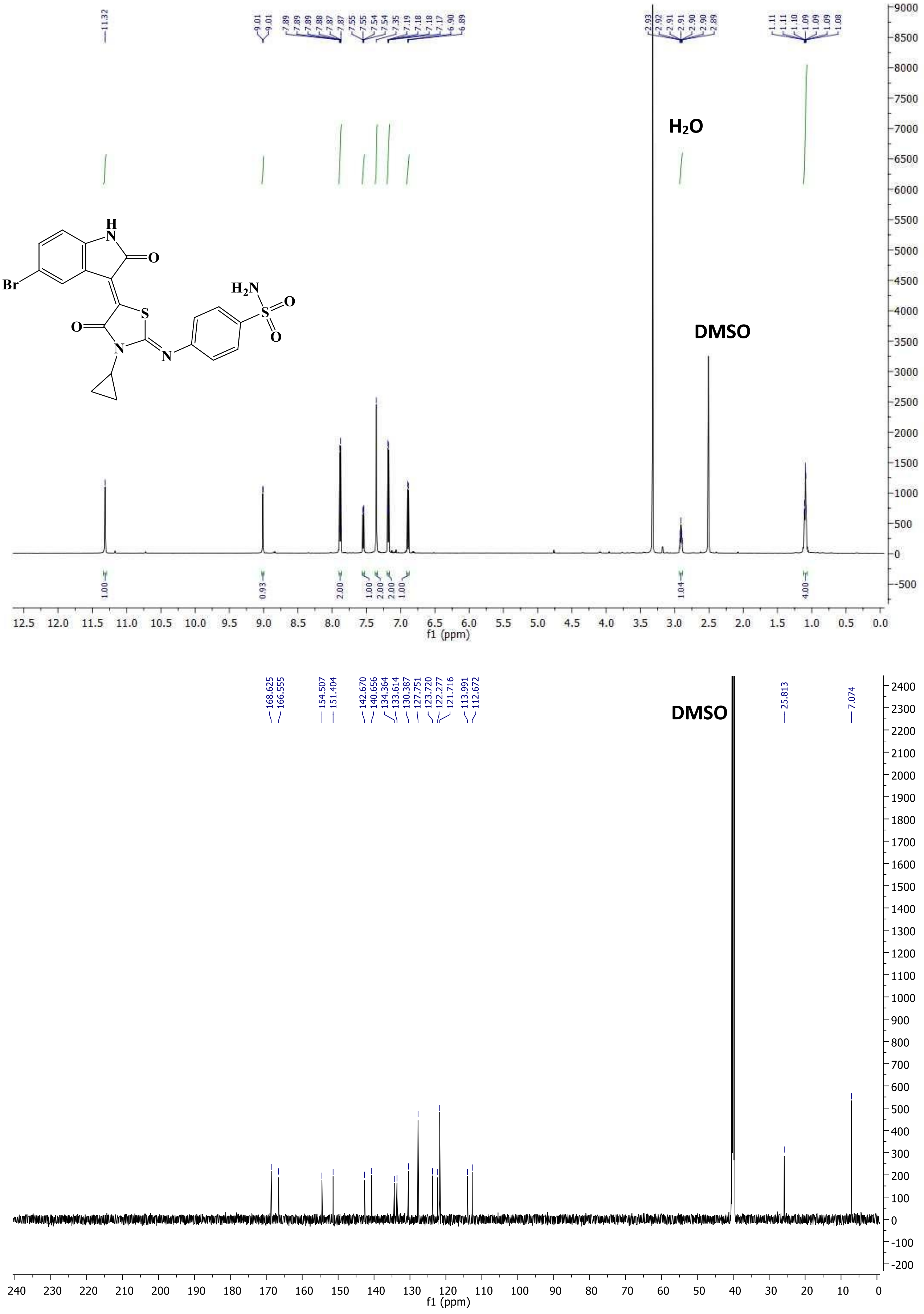

**Figure S7.** $^{1}H$ and $^{13}C$ NMR spectrum of 4-(((Z)-3-cyclopropyl-5-((Z)-5-methoxy-2-oxoindolin-3-ylidene)-4-oxothiazolidin-2-ylidene)amino)benzenesulfonamide **(3e)**

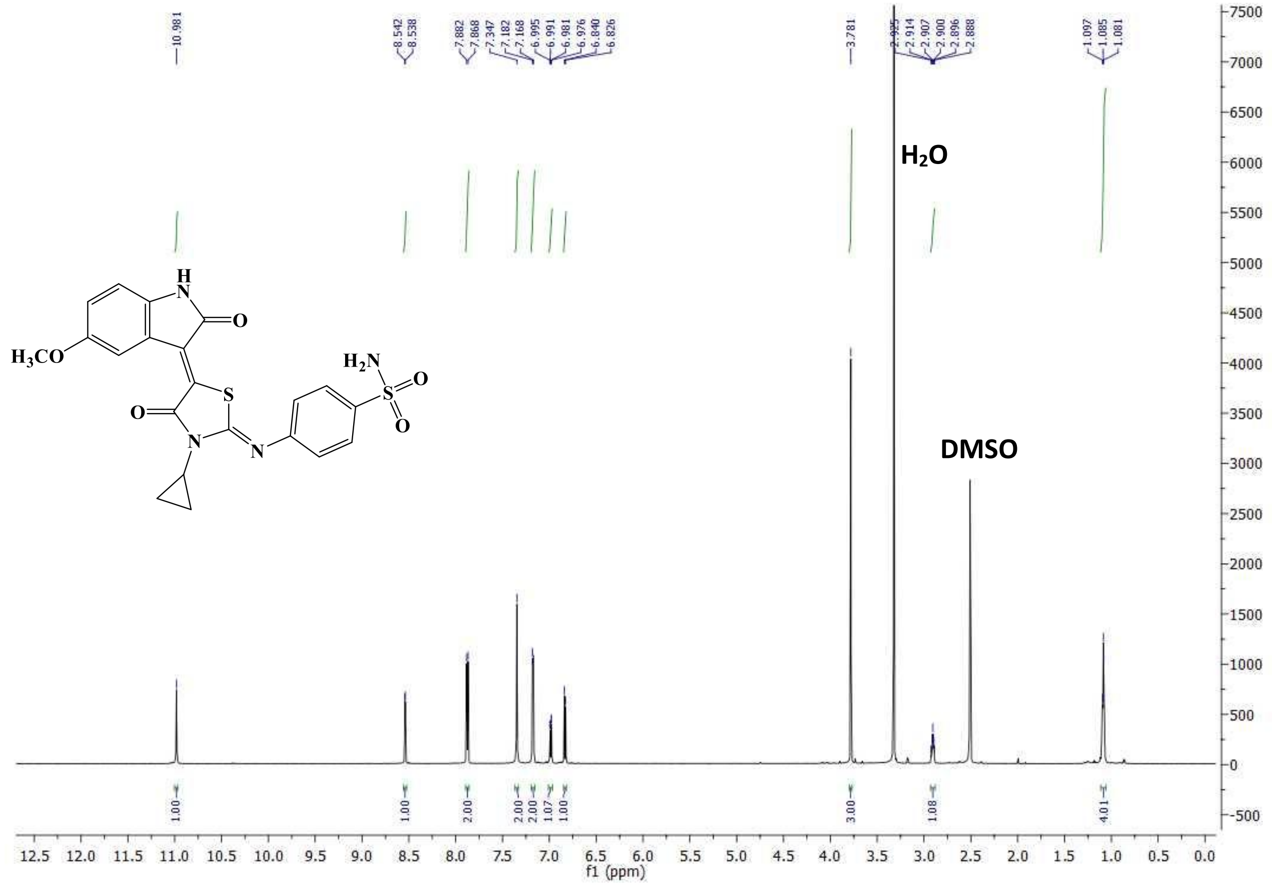


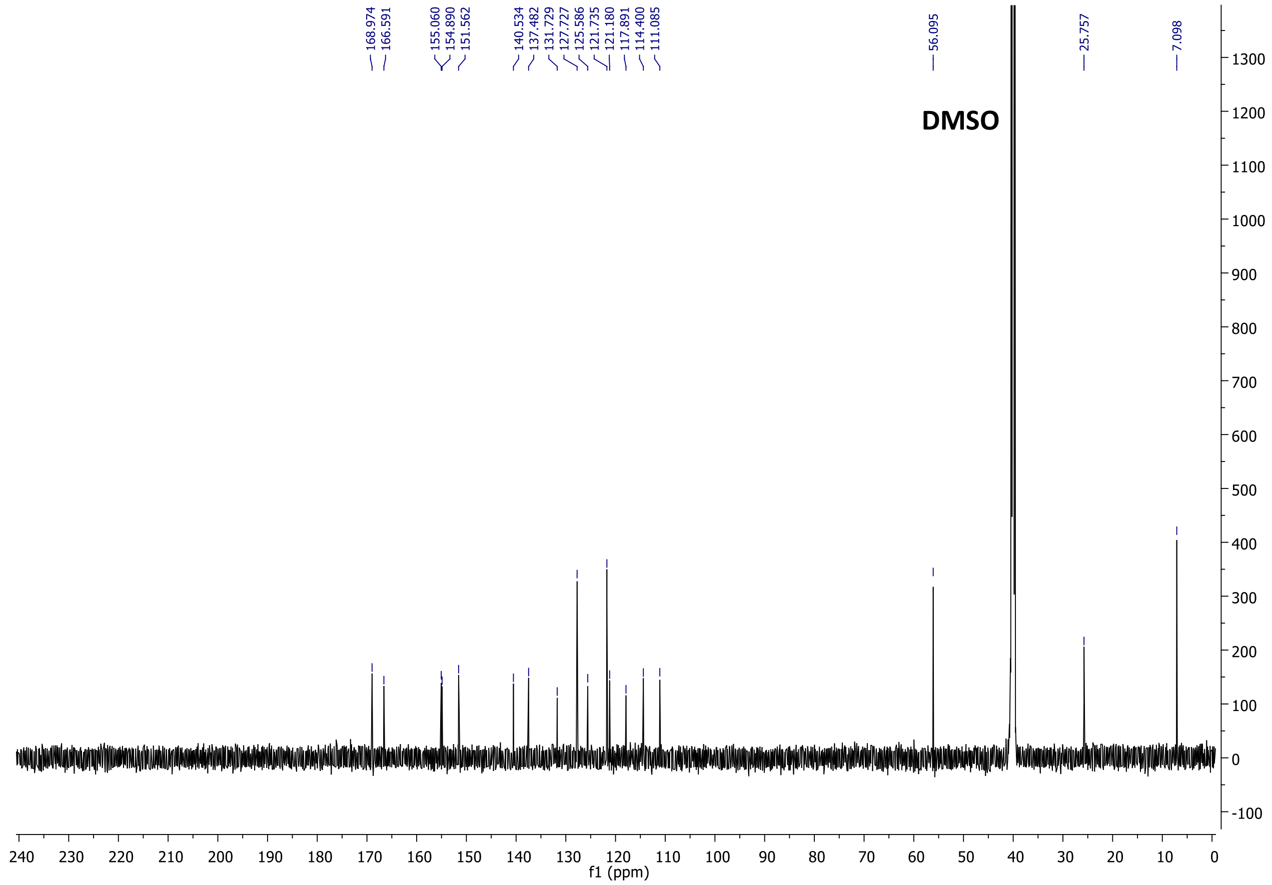

**Figure S8.** $^{1}H$ and $^{13}C$ NMR spectrum of 4-(((Z)-3-cyclopropyl-5-((Z)-5-methyl-2-oxoindolin-3-ylidene)-4-oxothiazolidin-2-ylidene)amino)benzenesulfonamide **(3f)**

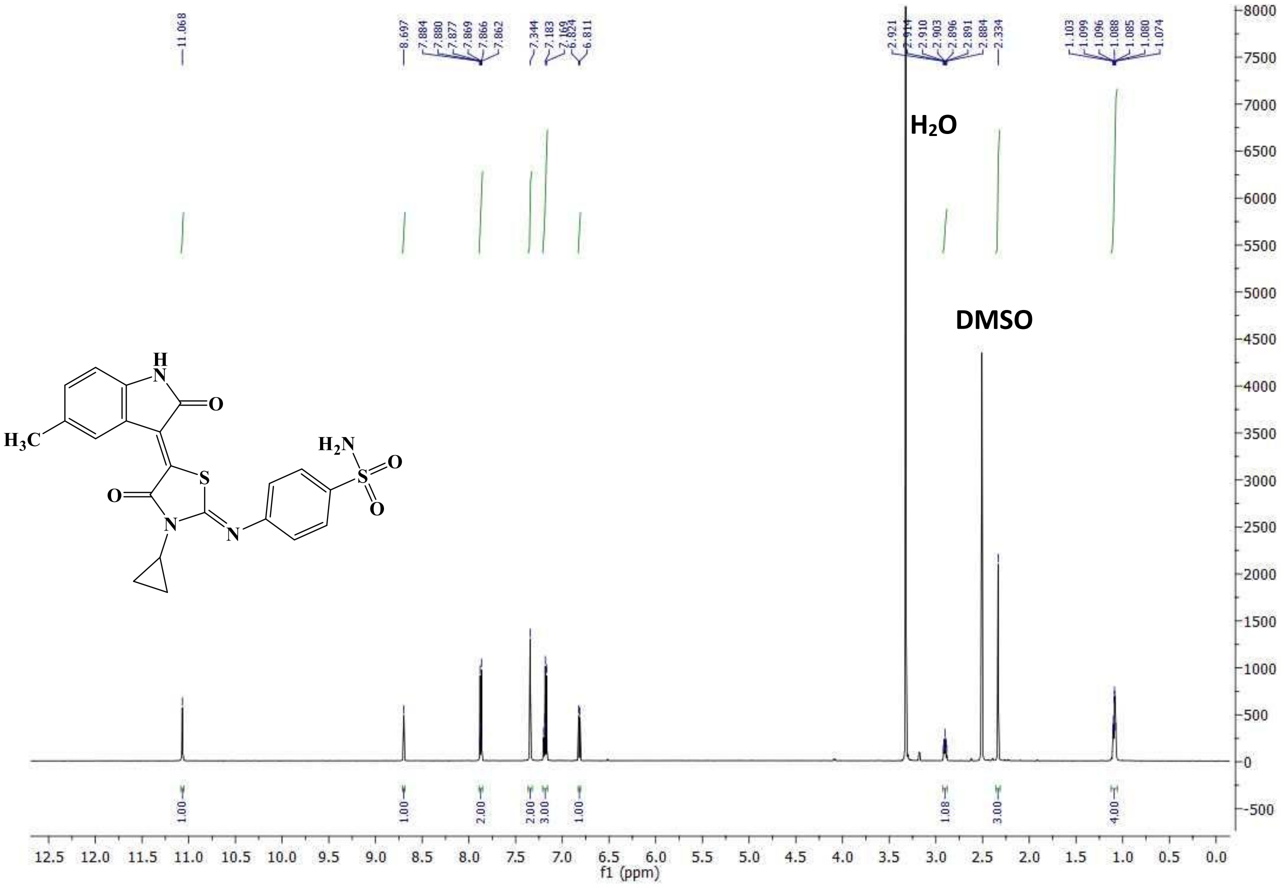


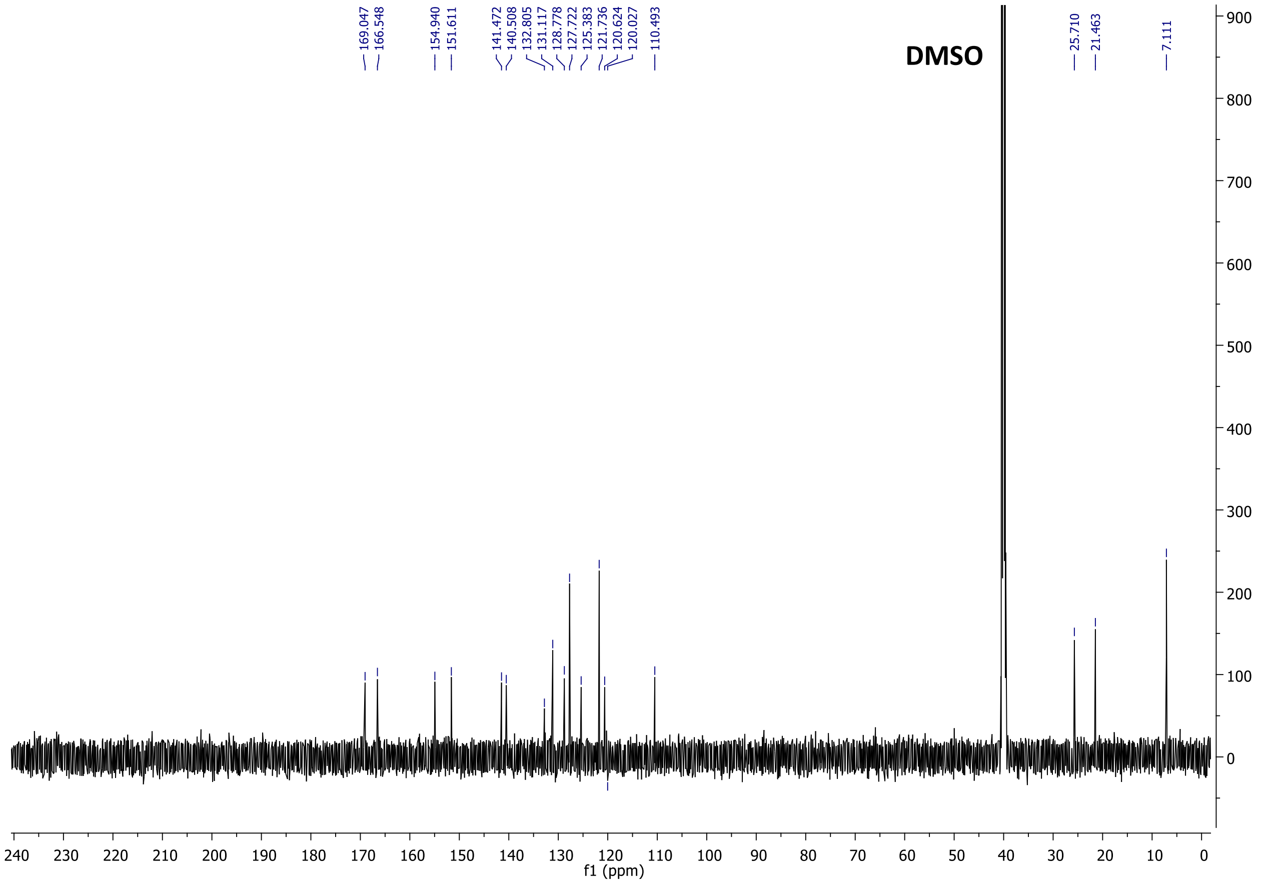

**Figure S9.** $^{1}H$ and $^{13}C$ NMR spectrum of 4-(((Z)-3-cyclopropyl-4-oxo-5-((Z)-2-oxoindolin-3-ylidene)thiazolidin-2-ylidene)amino)benzenesulfonamide **(3g)**

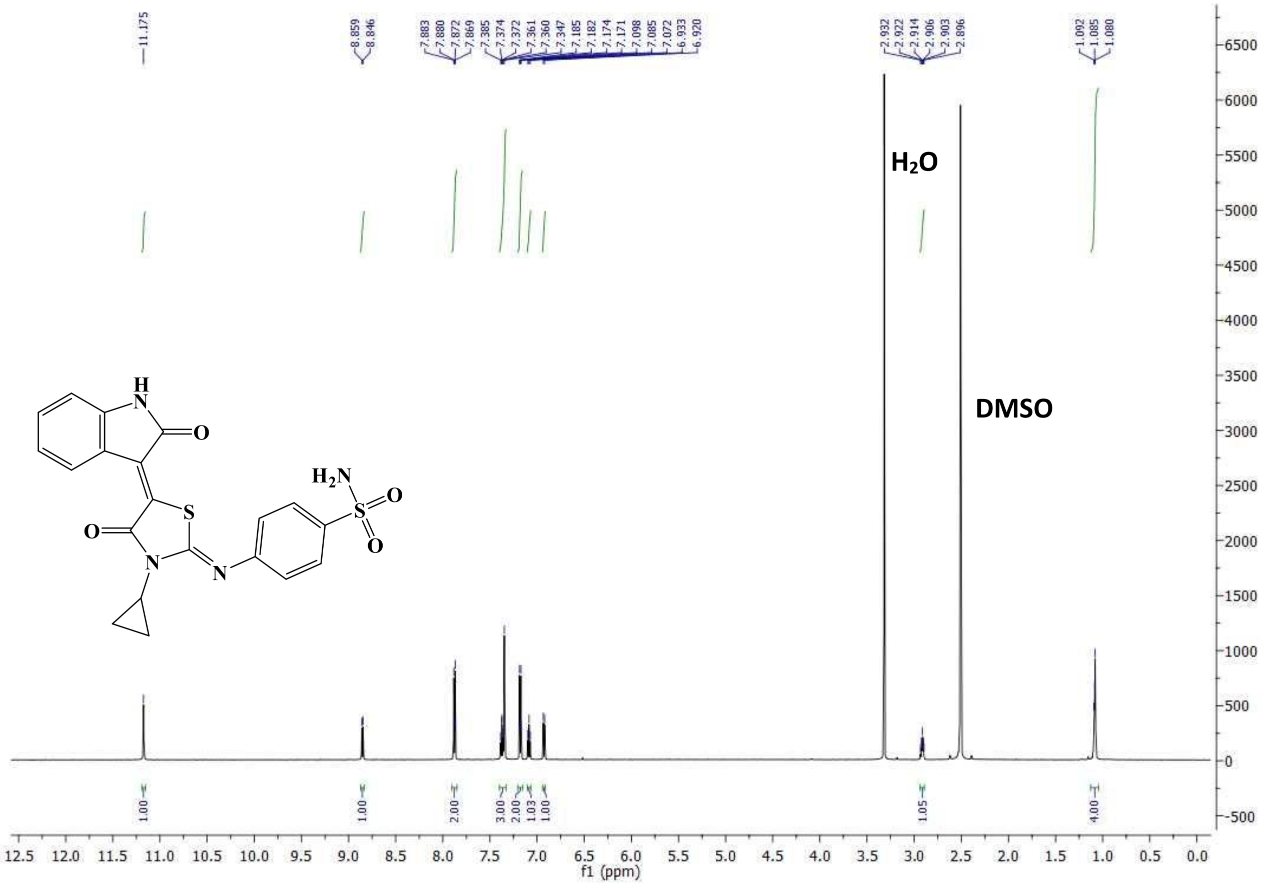


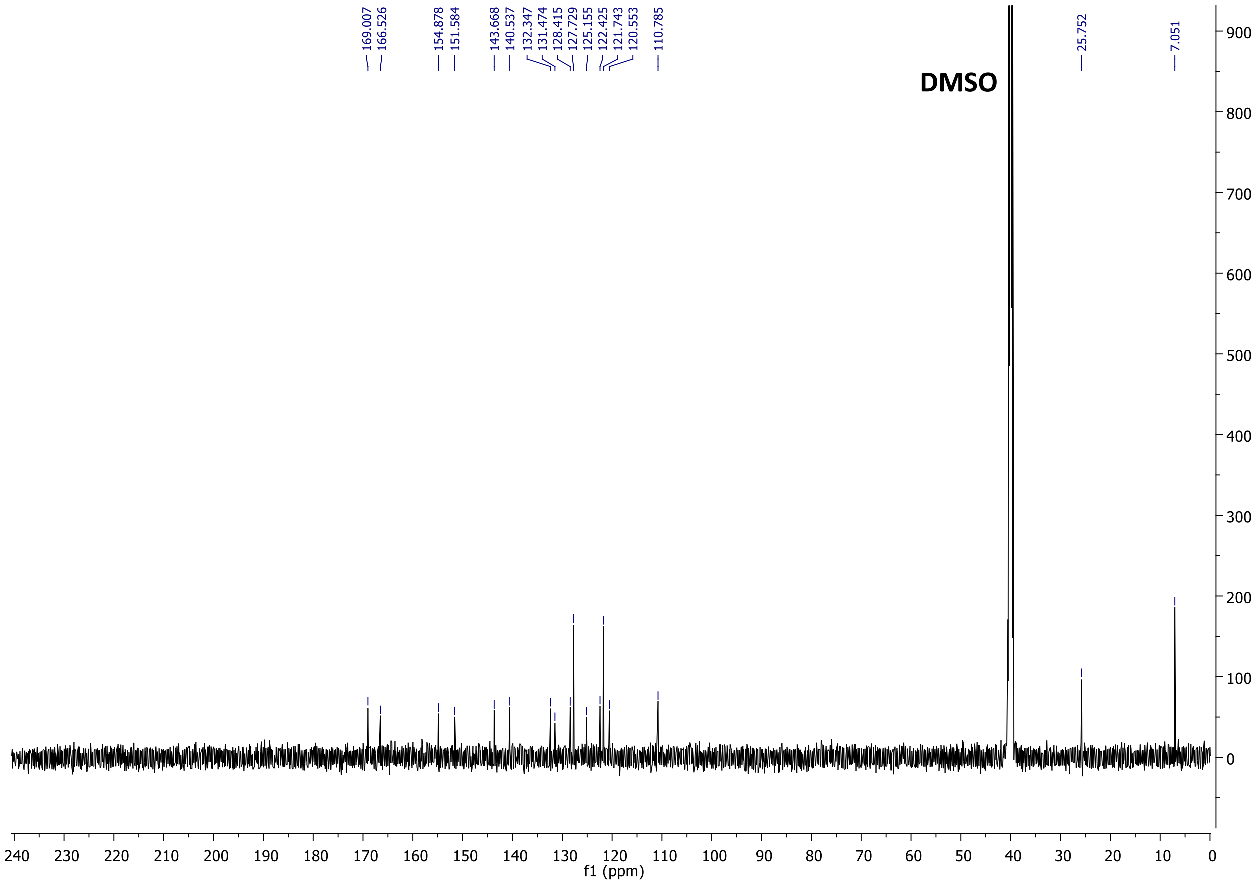

**Figure S10.** $^{1}H$ and $^{13}C$ NMR spectrum of 4-(((Z)-3-cyclopropyl-5-((Z)-7-fluoro-2-oxoindolin-3-ylidene)-4-oxothiazolidin-2-ylidene)amino)benzenesulfonamide **(3h)**

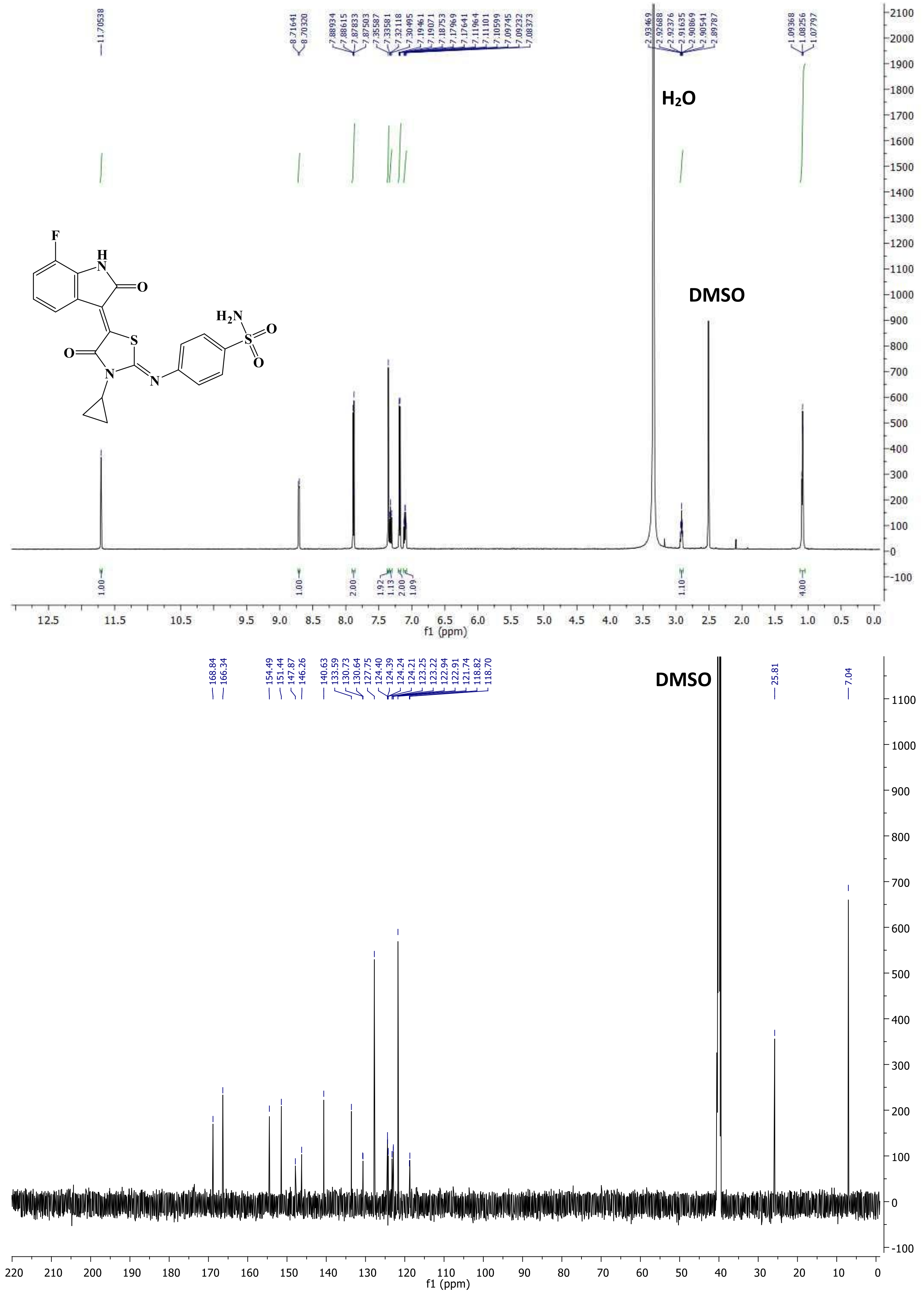

**Figure S11.** $^{1}H$ and $^{13}C$ NMR spectrum of 4-(((Z)-5-((Z)-7-bromo-2-oxoindolin-3-ylidene)-3-cyclopropyl-4-oxothiazolidin-2-ylidene)amino)benzenesulfonamide **(3i)**

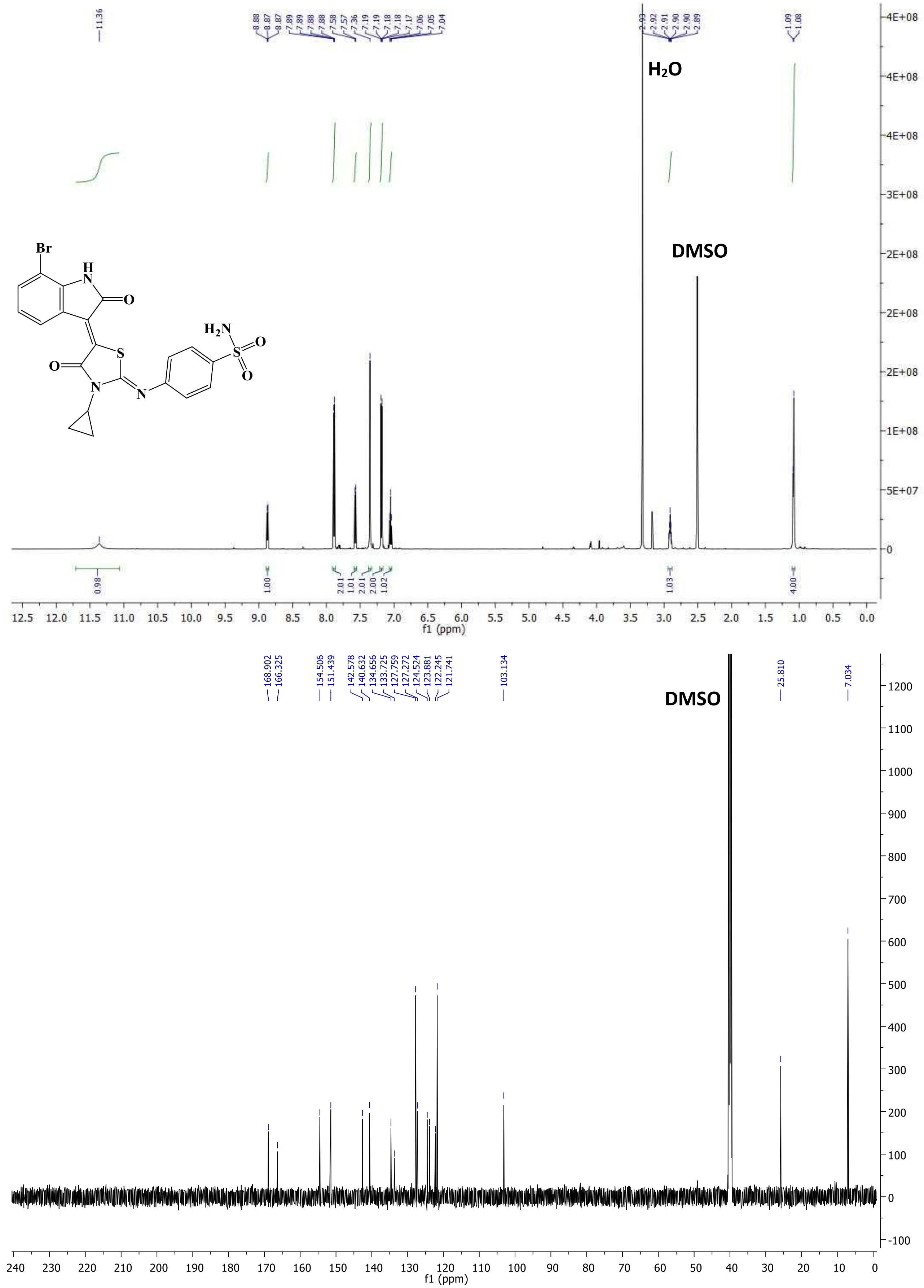

## 1.4. Mass spectra of compounds

**Figure S12.** HRMS spectrum of 4-(3-cyclopropylthioureido)benzenesulfonamide **(1)**

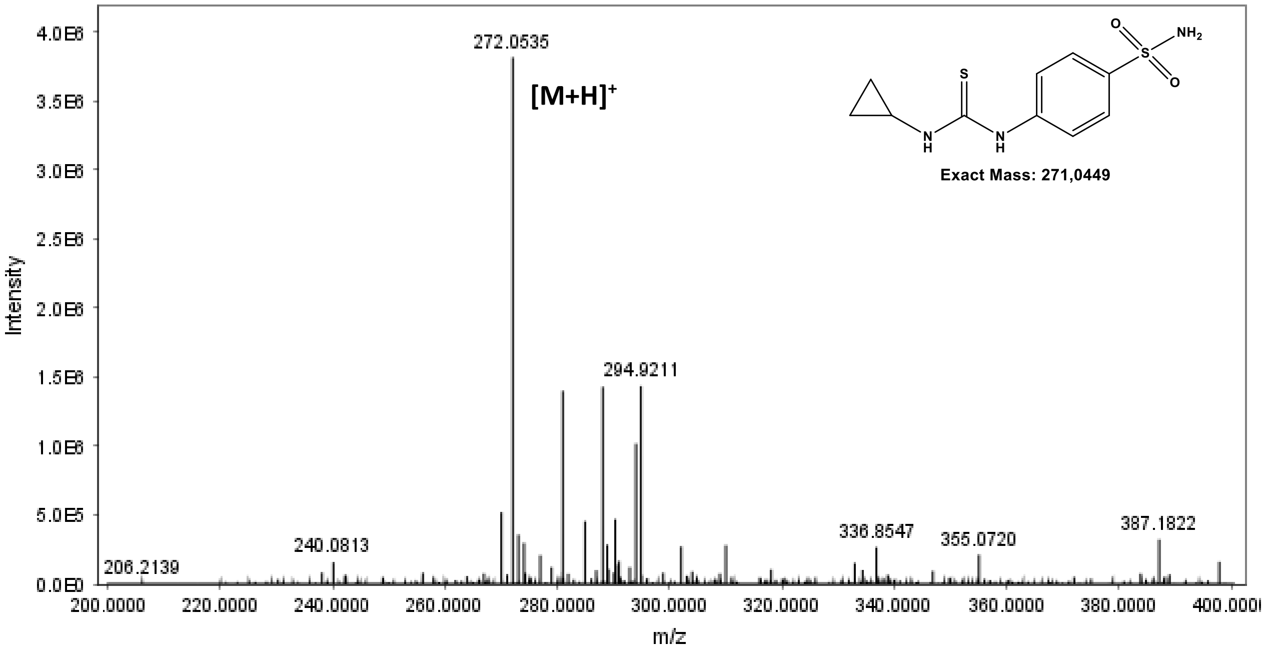


**Figure S13.** HRMS spectrum of (Z)-4-((3-cyclopropyl-4-oxothiazolidin-2-ylidene)amino)benzenesulfonamide **(2)**

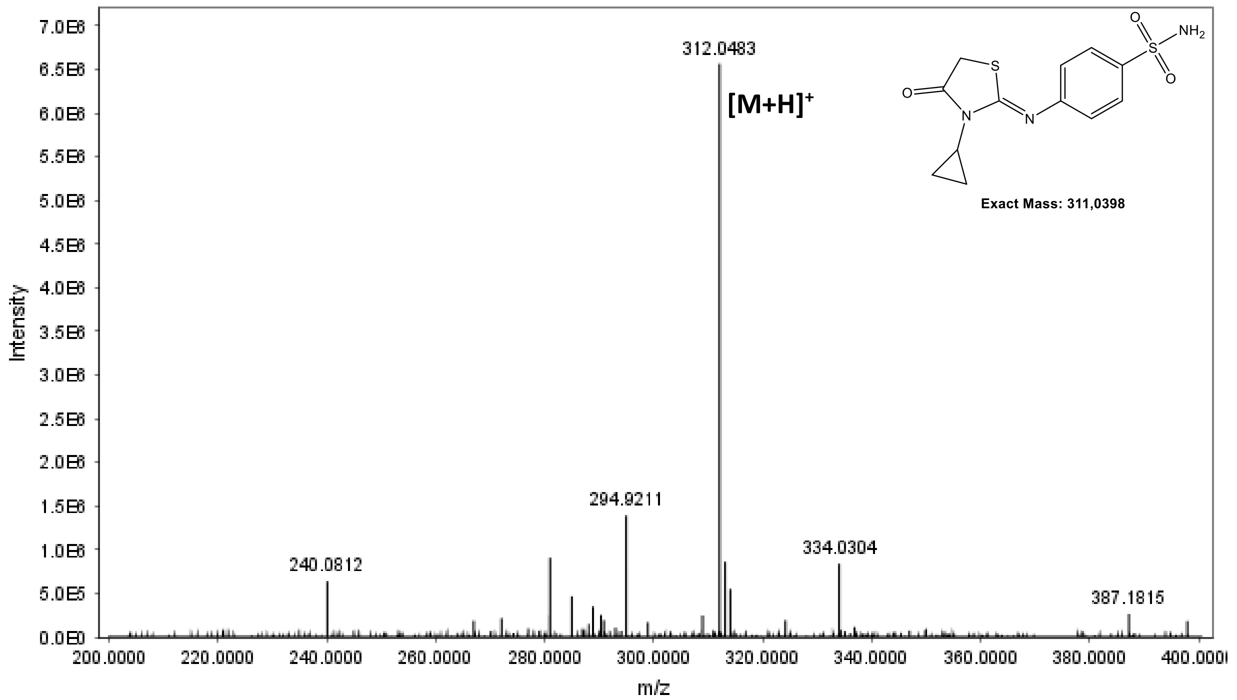

**Figure S14.** HRMS spectrum of 4-(((Z)-5-((Z)-5-chloro-2-oxoindolin-3-ylidene)-3-cyclopropyl-4-oxothiazolidin-2-ylidene)amino)benzenesulfonamide **(3a)**

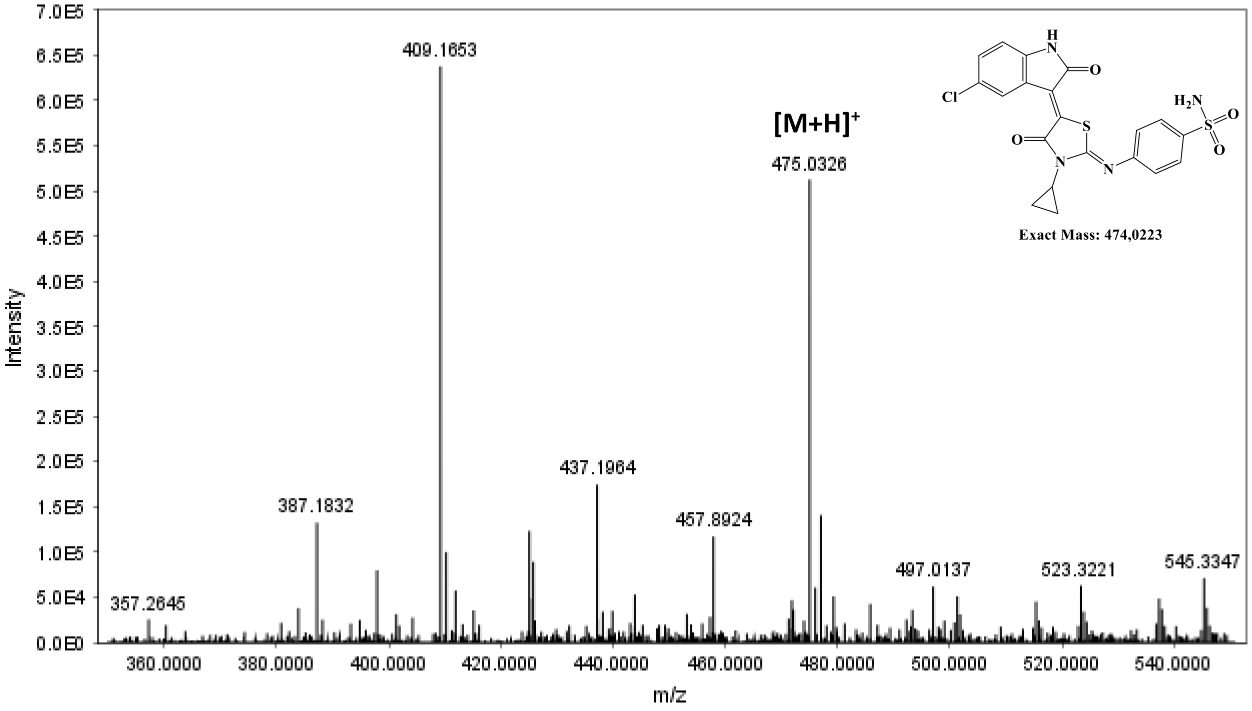


**Figure S15.** HRMS spectrum of 4-(((Z)-3-cyclopropyl-5-((Z)-5-nitro-2-oxoindolin-3-ylidene)-4-oxothiazolidin-2-ylidene)amino)benzenesulfonamide **(3b)**

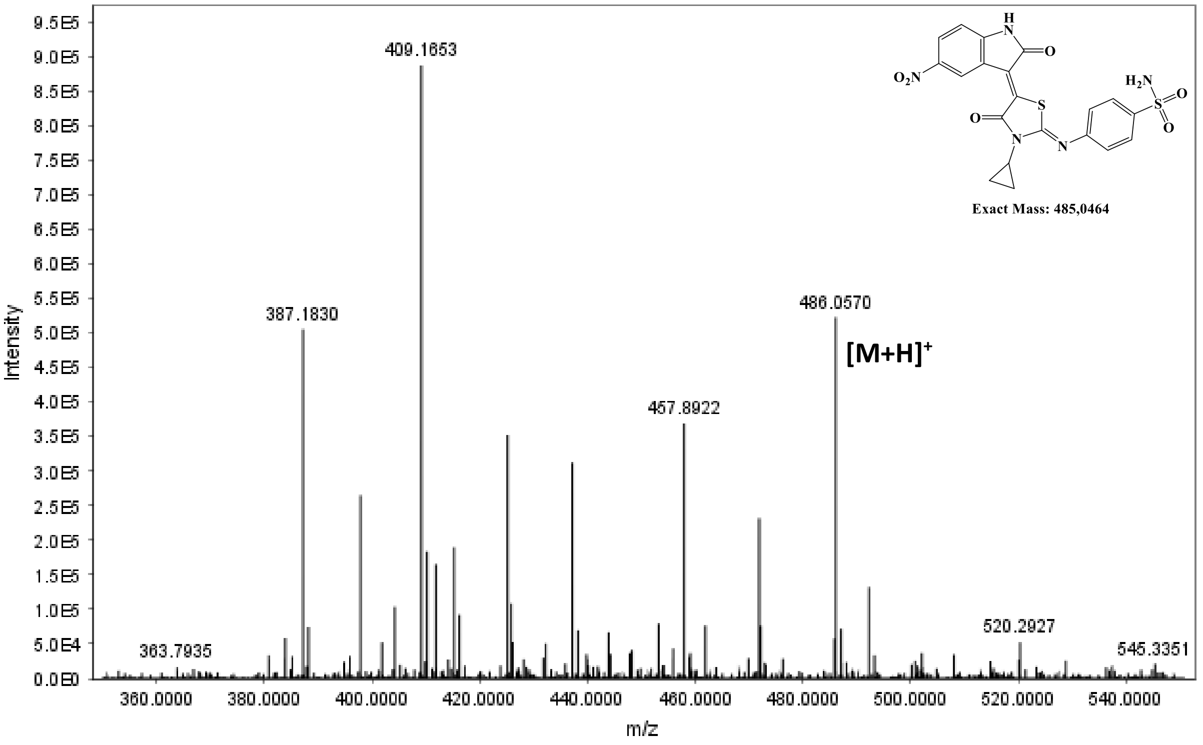

**Figure S16.** HRMS spectrum of 4-(((Z)-3-cyclopropyl-5-((Z)-5-fluoro-2-oxoindolin-3-ylidene)-4-oxothiazolidin-2-ylidene)amino)benzenesulfonamide **(3c)**

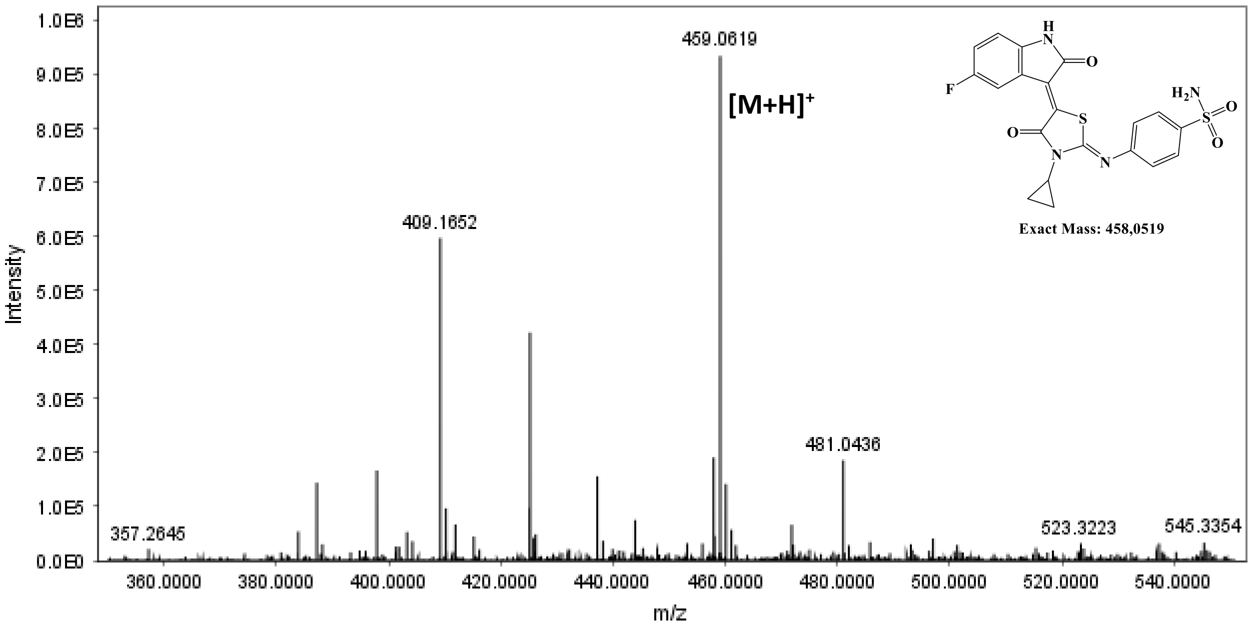


**Figure S17.** HRMS spectrum of 4-(((Z)-5-((Z)-5-bromo-2-oxoindolin-3-ylidene)-3-cyclopropyl-4-oxothiazolidin-2-ylidene)amino)benzenesulfonamide **(3d)**

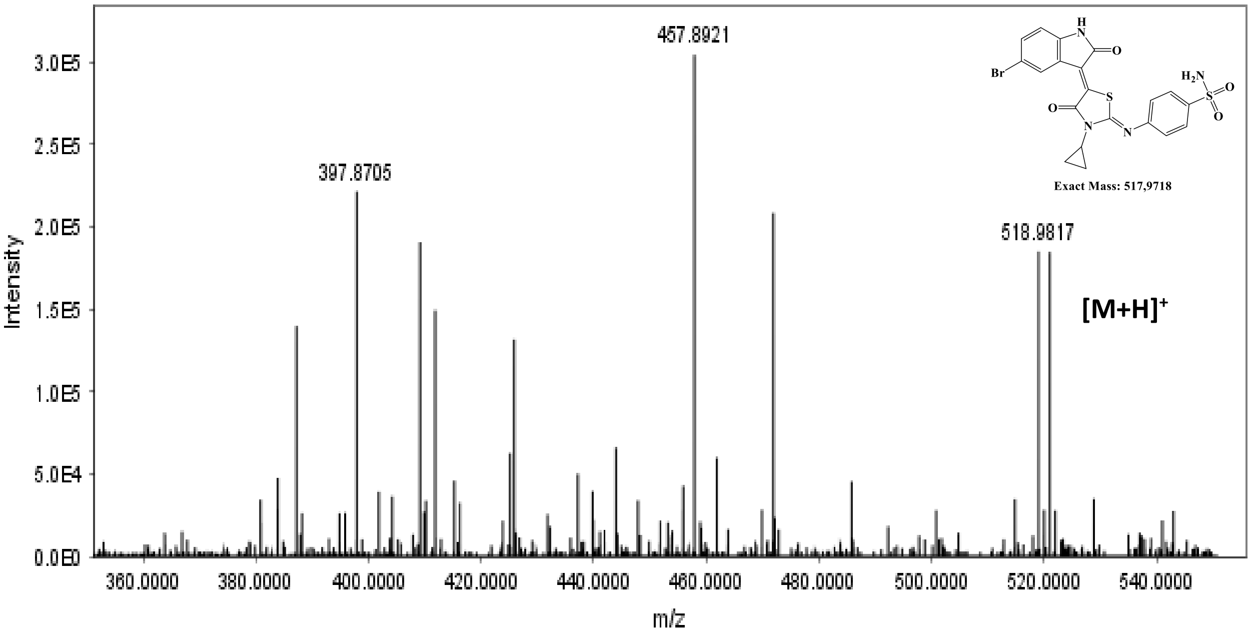

**Figure S18.** HRMS spectrum of 4-(((Z)-3-cyclopropyl-5-((Z)-5-methoxy-2-oxoindolin-3-ylidene)-4-oxothiazolidin-2-ylidene)amino)benzenesulfonamide **(3e)**

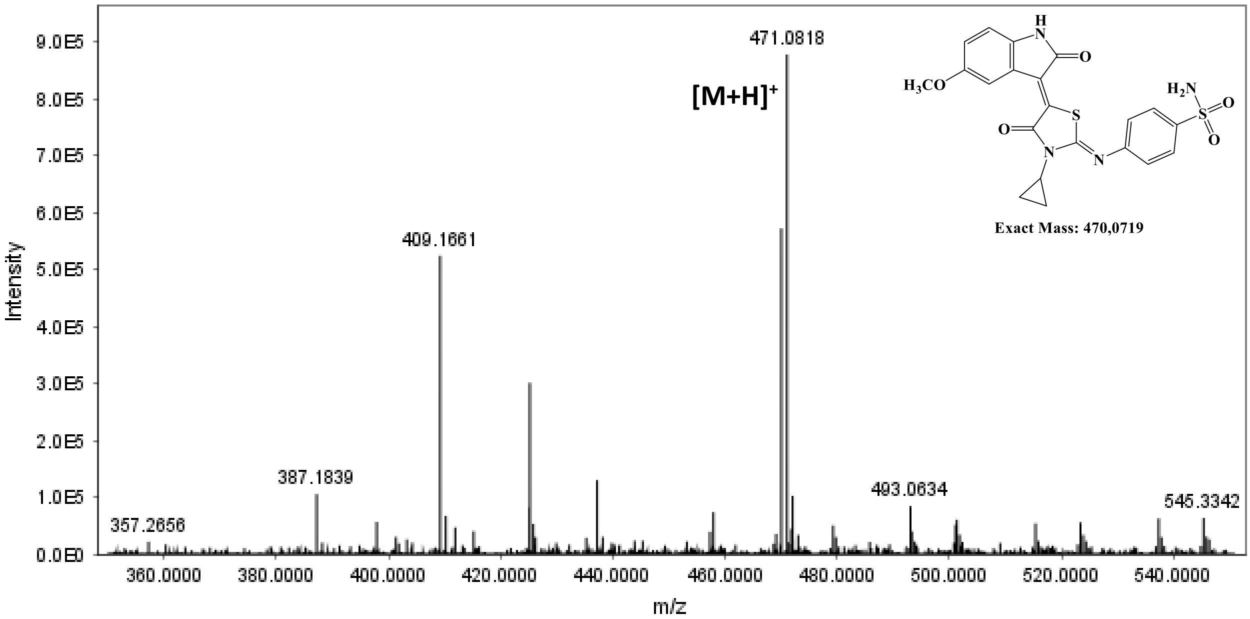


**Figure S19.** HRMS spectrum of 4-(((Z)-3-cyclopropyl-5-((Z)-5-methyl-2-oxoindolin-3-ylidene)-4-oxothiazolidin-2-ylidene)amino)benzenesulfonamide **(3f)**

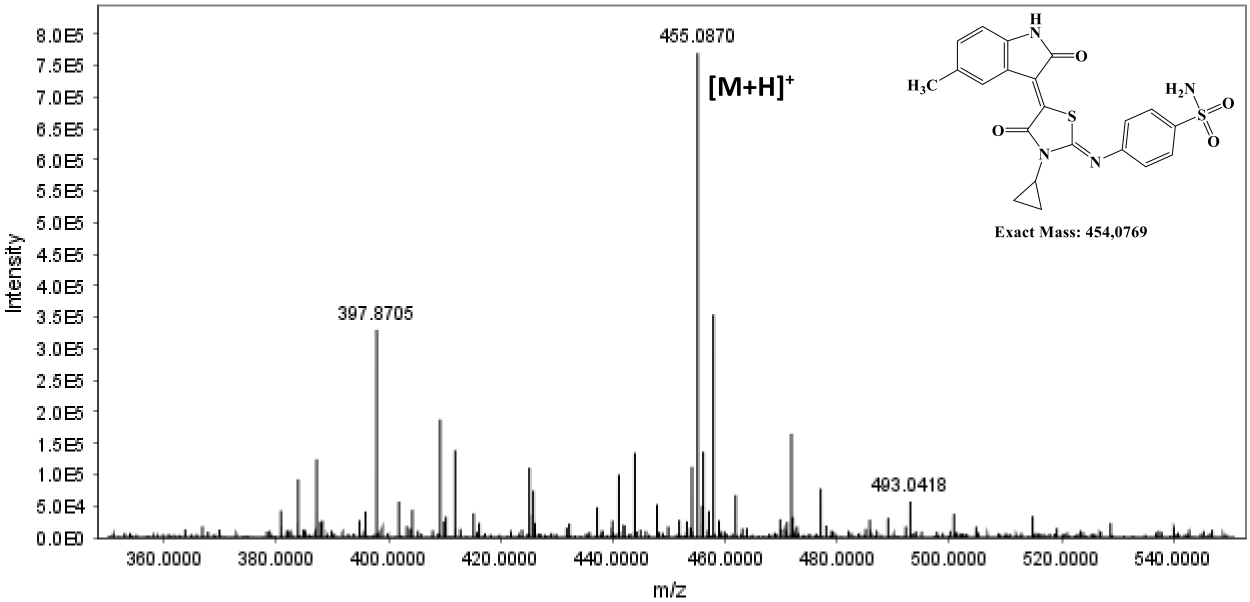

**Figure S20.** HRMS spectrum of 4-(((Z)-3-cyclopropyl-4-oxo-5-((Z)-2-oxoindolin-3-ylidene)thiazolidin-2-ylidene)amino)benzenesulfonamide **(3g)**

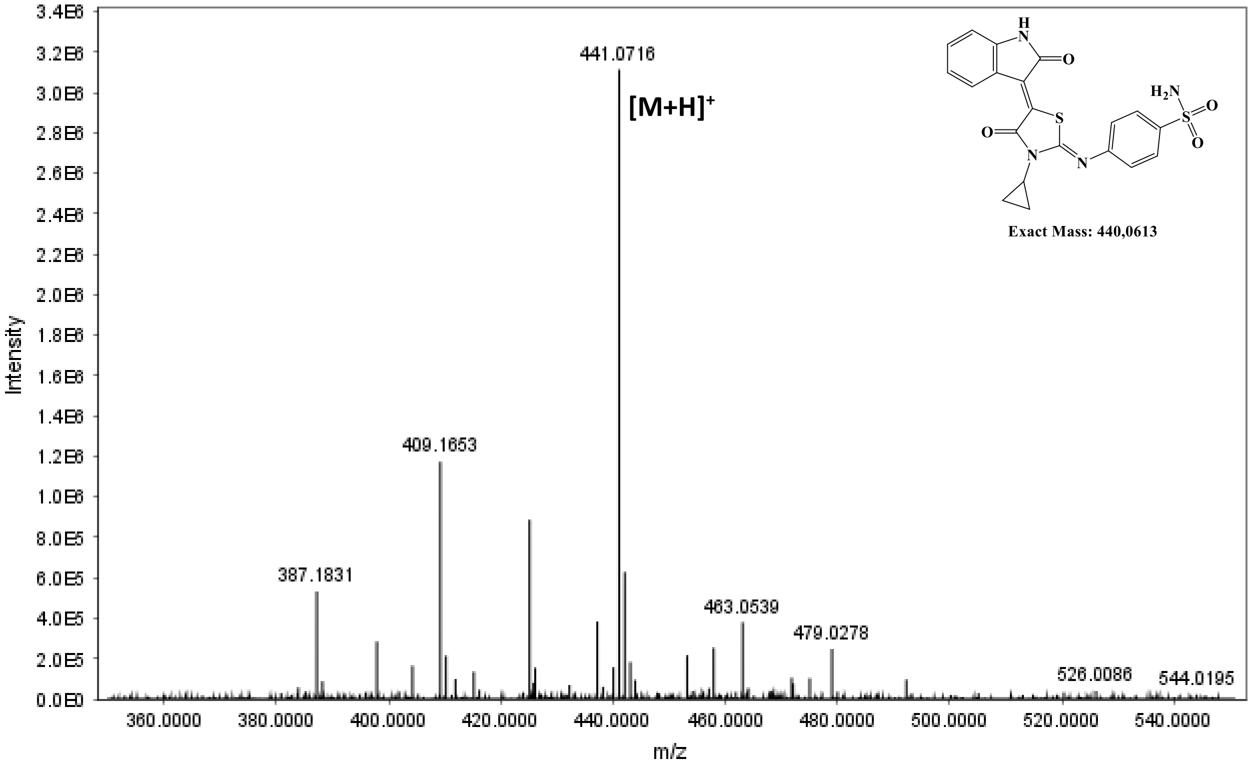


**Figure S21.** HRMS spectrum of 4-(((Z)-3-cyclopropyl-5-((Z)-7-fluoro-2-oxoindolin-3-ylidene)-4-oxothiazolidin-2-ylidene)amino)benzenesulfonamide **(3h)**

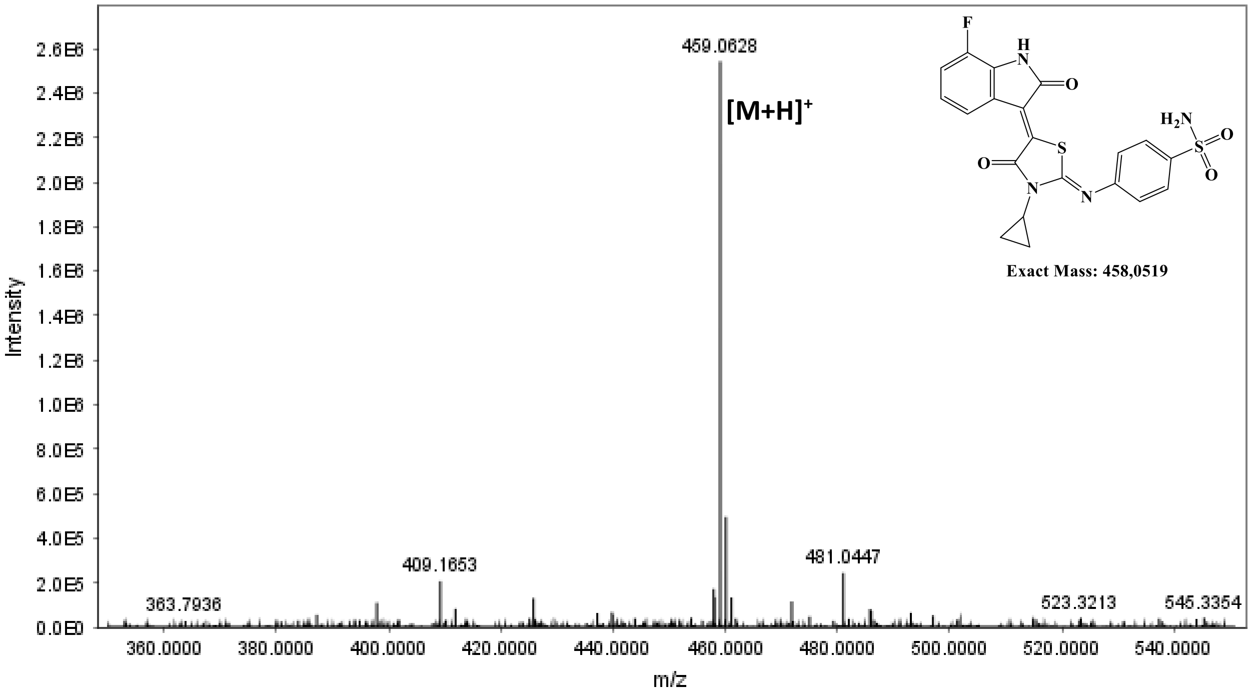

**Figure S22.** HRMS spectrum of 4-(((Z)-5-((Z)-7-bromo-2-oxoindolin-3-ylidene)-3-cyclopropyl-4-oxothiazolidin-2-ylidene)amino)benzenesulfonamide **(3i)**

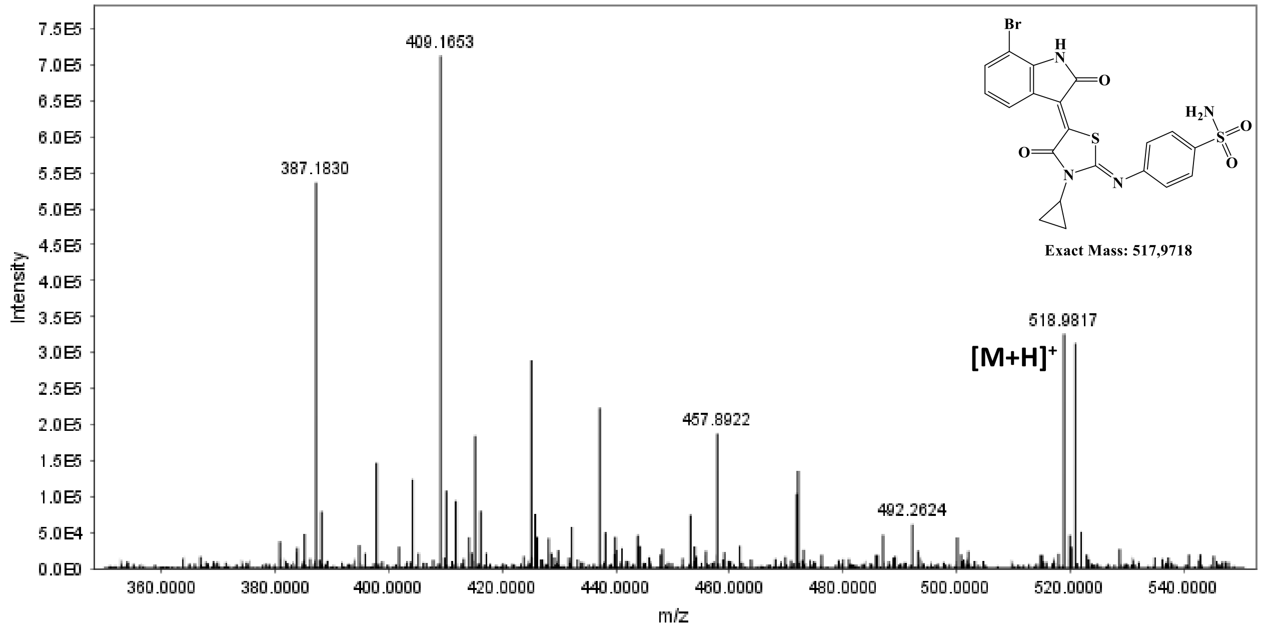


## 1.5. X-Ray Cristallography

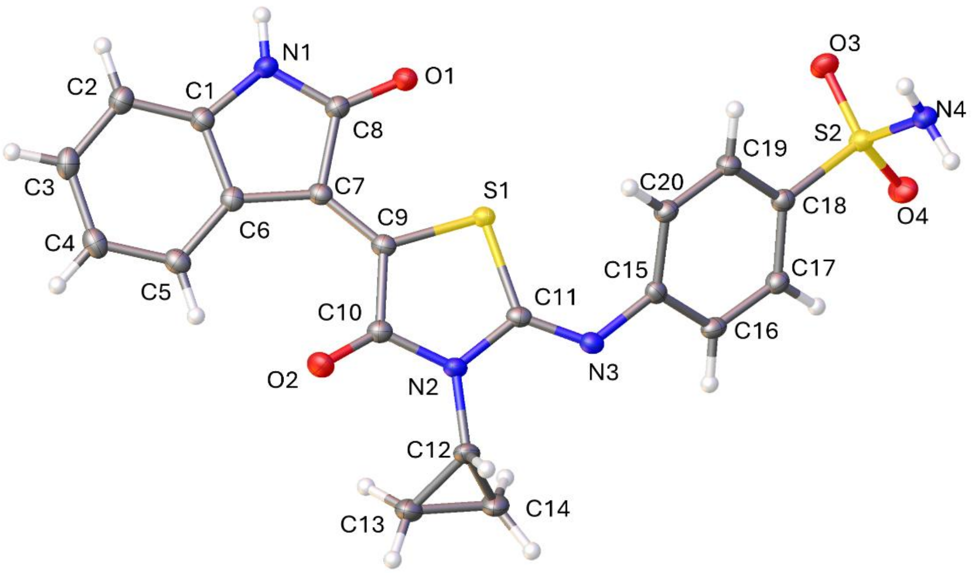


**Figure S23.** Single crystal X-ray diffraction structure of compound **3g** in ZZ configuration.

## 2. Molecular modelling

### 2.1. Ligands and protein preparation

All ligands were sketched and prepared using the Maestro Graphical User Interface (GUI) *ver*. 2024-1 [6]. Epik module was used to calculate the protonation state at pH 7.4 in water and to obtain the most stable conformation and molecular mechanics conformational analysis was performed using MacroModel software [7]. For the calculations, Merck Molecular Force Field (MMFF) was set as a *force field* and solvent effects by adopting the generalised Born/Surface Area (GB/SA) water implicit solvation model [8] [9]. The simulations were performed with a 5000-step Monte Carlo calculation using the Polak-Ribier Conjugate Gradient (PRCG) method, and a convergence criterion of 0.05 kcal/mol was used. All other parameters remained at their standard values.

The protein complex three-dimensional (3D) coordinates of hCA II (3HS4), hCA IX (3IAI) and hCA XII (1JD0) were obtained from the Protein Data Bank (PDB) website (www.rcsb.org). All 3D structures were optimised using the Protein Preparation Wizard (PPW) tool [6], implemented in the Maestro GUI, to assign bond orders, add hydrogen atoms, adjust disulfide bonds, and assign residues protonation state at pH 7.4. After refinement, 3HS4, 3IAI, and 1JD0 were submitted to 10,000 steps of MacroModel energy minimisation using the OPLS_2005 force field.

### 2.2. Molecular docking

A two-step docking study was performed using the Glide tool [10] and refined with Induced Fit Docking (IFD) calculations [11]. At first, the ligands were extracted from the selected crystallographic structures to validate the docking protocol and re-docked into their respective original binding sites. The ligand coordinates were used to center the box for the docking simulations. First, the SP and XP protocols were chosen to assess which one could better reproduce the crystallographic poses. This process was carried out for both minimized and non-minimized 3D hCAII/IX/XII structures. By examining the Root Mean Square Deviation (RMSD) results calculated on the ligand-heavy atoms, the Standard Precision (SP) protocol effectively replicated the co-crystallized pose seen experimentally (Table S1).

Secondly, the docking results were further optimized using IFD calculation in "*standard sampling*" (20 poses). After visual inspection, the Molecular Mechanics Generalized Born/Surface Area (MM-GBSA) [12] [13] method was applied to compute the binding free energies (ΔG bind) for the best IFD pose.

All images were rendered using the Maestro GUI suite 2024-1.

**Table S1.** Docking protocol validation.

| STEP1 | | PDB | LIG | SP | | | XP | | |
|---|---|---|---|---|---|---|---|---|---|
| | | Res. | | G-Score* | RMSD | MMGBSA* | G-Score* | RMSD | MMGBSA* |
| ***h*CA II** | 3HS4 | 1.10 Å | AAZ | -6.23 | 0.54 | -39.04 | -5.71 | 2.27 | -46.84 |
| | 3HS4-min | | | -6.83 | 0.70 | -42.62 | -5.31 | 3.40 | -35.59 |
| ***h*CA IX** | 3IAI | 2.20 Å | AAZ | -5.48 | 2.64 | -23.31 | -5.66 | 4.43 | -33.87 |
| | 3IAI-min | | | -7.59 | 1.66 | -38.09 | -5.35 | 4.22 | -34.42 |
| ***h*CA XII** | 1JD0 | 1.50 Å | AAZ | -6.40 | 1.03 | -35.23 | -5.34 | 2.98 | -32.95 |
| | 1JD0-min | | | -6.30 | 1.01 | -40.02 | -5.9 | 3.7 | -34.47 |

*kcal/mol

As a further investigation, the atomic distances between **3h** and the receptor were measured in terms of the distance between the sulfonamide moiety and $Zn^{2+}$. In the case of hCA II, the distance is 6.67 Å, which is too large to allow the $Zn^{2+}$ binding (Figure S24, panel A) while in hCA IX and XII the distance is shorter, 2.43 Å and 2.38 Å, respectively (Figure S24, panels B and C).

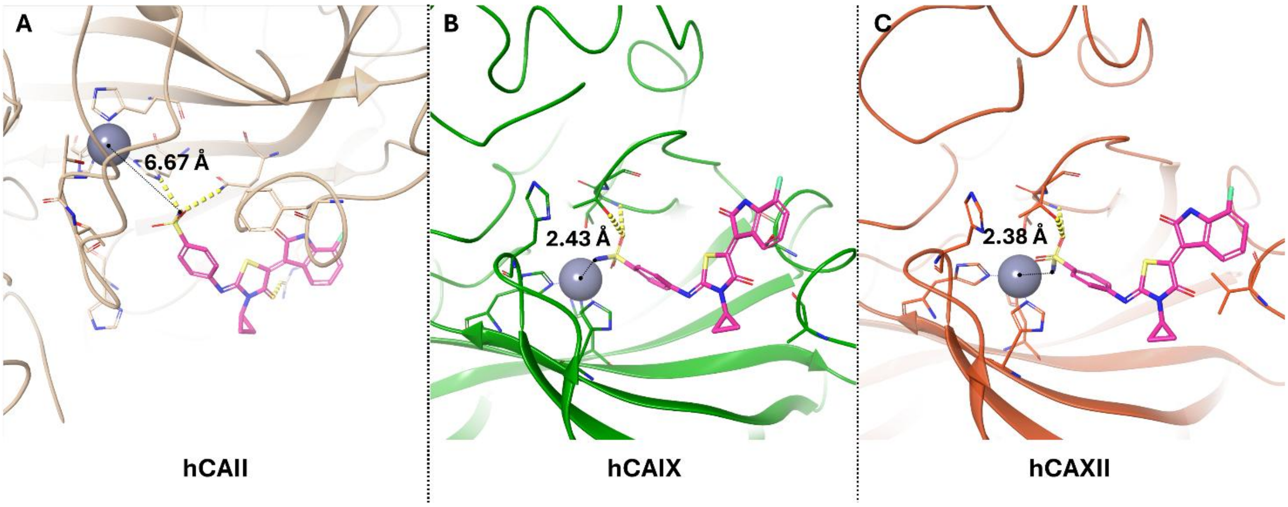


**Figure S24.** Distances between the -NH group of the sulfonamide moiety and $Zn^{2+}$. A) hCA II: 6.67 Å (large distance, weak interaction); (B) hCA IX: 2.43 Å (short distance, strong interaction); (C) hCA XII: 2.38 Å (short distance, strong interaction). Shorter distances in hCAIX and XII suggest higher inhibitory potency.

## 2.3. QikProp prediction of drug-like properties

An *in silico* ADMET properties prediction was carried out to assess the drug-like properties of the synthesised compounds. This comprehensive evaluation, performed using QikProp software [14], focused on a on a set of carefully selected critical properties described in Table S2.

**Table S2.** QikProp properties with description and range or recommended values.

| Property or Descriptor | Description | Range or Recommended Values |
|---|---|---|
| Stars | Number of property or descriptor values that fall outside the 95% range of similar values for known drugs. A large number of stars suggests that a molecule is less drug-like than molecules with few stars. The following properties and descriptors are included in the determination of stars: MW, dipole, IP, EA, SASA, FOSA, FISA, PISA, WPSA, PSA, volume, rotor, donorHB, accptHB, glob, QPpolrz, QPlogPC16, QPlogPoct, QPlogPw, QPlogPo/w, logS, QPLogKhsa, QPlogBB, metabol. | 0 – 5 |
| mol_MW | Molecular weight of the molecule. | 130.0 – 725.0 |
| HBD | Estimated number of hydrogen bonds that would be donated by the solute to water molecules in an aqueous solution. Values are averages taken over a number of configurations, so they can be non-integer. | 0.0 – 6.0 |
| HBA | Estimated number of hydrogen bonds that would be accepted by the solute from water molecules in an aqueous solution. Values are averages taken over a number of configurations, so they can be non-integer. | 2.0 – 20.0 |
| QPlogPo/w | Predicted octanol/water partition coefficient. | -2.0 – +6.5 |
| Rotor | Number of non-trivial (not CX3), non-hindered (not alkene, amide, small ring) rotatable bonds. | 0 – 15 |
| PSA | Van der Waals surface area of polar nitrogen and oxygen atoms. | 7.0 – 200.0 |
| QPlogS | Predicted aqueous solubility, log S. S in mol dm–3 is the concentration of the solute in a saturated solution that is in equilibrium with the crystalline solid. | -6.5 – +0.5 |
| QPPCaco | Predicted apparent Caco-2 (colorectal adenocarcinoma) cell permeability in nm/sec. Caco-2 cells are a model for the gut-blood barrier. QikProp predictions are for non-active transport. | < 25: poor<br>> 500: great |
| QPPMDCK | Predicted apparent MDCK (Madin-Darby Canine Kidney) cell permeability in nm/sec. MDCK cells are considered to be a good mimic for the blood-brain barrier. QikProp predictions are for non-active transport. | < 25: poor<br>> 500: great |
| % OA | Predicted human oral absorption on 0 to 100% scale. The prediction is based on a quantitative multiple linear regression model. This property usually correlates well with HumanOral-Absorption, as both measure the same property. | > 80%: high<br>< 25%: poor |
| QPlogHERG | Predicted $IC_{50}$ value for blockage of HERG K+ channels. | Concern below -5 |
| CNS | Predicted central nervous system activity on a –2 (inactive) to +2 (active) scale. | -2 – +2 |
| QPlogBB | Predicted brain/blood partition coefficient. Note: QikProp predictions are for orally delivered drugs so, for example, dopamine and serotonin are CNS negative because they are too polar to cross the blood-brain barrier | -3 – +1.2 |
| QPlogKhsa | Prediction of binding to human serum albumin. | -1.5 – +1.5 |

It is noteworthy that all compounds were assigned a rating between 0 and 3 stars, indicating that the majority fell within acceptable ranges for almost all key properties.

In Table S3 the properties considered in Lipinski's Rule of Five (RO5) and other relevant physico-chemical properties, such as polar surface area (PSA) e solubility (S) are reported. In Table S4 additional properties that influence the pharmacokinetics and safety of the compounds are illustrated.

**Table S3.** Predicted properties for the series **3a-i**.

| Molecule | #stars | mol MW | HBD | HBA | QPlogPo/w | #rotor | PSA | QPlogS |
|---|---|---|---|---|---|---|---|---|
| | 0 – 5 | 130 – 725 | 0 – 6 | 2 – 20 | -2 – 6.5 | 0 – 15 | 7 – 200.0 | -6.5 – 0.5 |
| 3a | 1 | 474.936 | 3 | 11 | 1.503 | 4 | 140.925 | -5.398 |
| 3b | 3 | 485.488 | 3 | 12 | 0.366 | 5 | 185.768 | -4.791 |
| 3c | 1 | 458.481 | 3 | 11 | 1.263 | 4 | 140.924 | -5.059 |
| 3d | 1 | 519.387 | 3 | 11 | 1.575 | 4 | 140.927 | -5.497 |
| 3e | 1 | 470.517 | 3 | 11.75 | 1.121 | 5 | 149.214 | -4.901 |
| 3f | 1 | 454.518 | 3 | 11 | 1.311 | 4 | 140.939 | -5.217 |
| 3g | 1 | 440.491 | 3 | 11 | 1.04 | 4 | 140.935 | -4.721 |
| 3h | 1 | 458.481 | 3 | 11 | 1.298 | 4 | 140.841 | -5.159 |
| 3i | 1 | 519.387 | 3 | 11 | 1.61 | 4 | 140.347 | -5.623 |

**Table S4.** Predicted pharmacokinetics properties for the series **3a-i**.

| Molecule | QPPCaco | QPPMDCK | %OA | QPlogHERG | CNS | QPlogBB | QPlogKhsa |
|---|---|---|---|---|---|---|---|
| | < 25 poor >500 great | < 25 poor >500 great | > 80 % is high < 25 % is poor | >-5 | - 2 (inactive) to +2 (active) | -3.0 – 1.2 | -1.5 – 1.5 |
| 3a | 43.647 | 52.909 | 65.099 | -6.211 | -2 | -2.137 | -0.185 |
| 3b | 5.875 | 2.466 | 29.895 | -6.17 | -2 | -3.362 | -0.329 |
| 3c | 43.738 | 38.869 | 63.708 | -6.177 | -2 | -2.163 | -0.242 |
| 3d | 43.71 | 57.039 | 52.569 | -6.234 | -2 | -2.133 | -0.167 |
| 3e | 43.838 | 21.673 | 62.895 | -6.183 | -2 | -2.375 | -0.28 |
| 3f | 43.932 | 21.663 | 64.025 | -6.222 | -2 | -2.329 | -0.153 |
| 3g | 43.932 | 21.663 | 64.025 | -6.222 | -2 | -2.329 | -0.153 |
| 3h | 43.872 | 40.657 | 63.94 | -6.258 | -2 | -2.183 | -0.238 |
| 3i | 43.687 | 58.396 | 52.774 | -6.358 | -2 | -2.171 | -0.163 |

# 3. Biological evaluation

## 3.1. Carbonic anhydrase inhibition assay

The CA catalyzed $CO_2$ hydration/inhibition was measured using a stopped-flow instrument as previously described method [15]. Initial rates of the CA-catalyzed $CO_2$ hydration reactions were followed for 10–100s. The $CO_2$ concentrations ranged from 1.7 to 17 mM for the determination of the inhibition constants. For each inhibitor, at least six traces of the initial 5–10% of the reaction were used to assess the initial velocity. The uncatalyzed rates were subtracted from the total observed rates. Stock solutions of inhibitors (10 mM) and dilutions up to 0.01 nM were prepared in distilled-deionized water. Inhibitor and enzyme solutions were preincubated together for 15 min at room temperature prior to assay, to allow for the formation of the E–I complex. The inhibition constants were obtained by non-linear least-squares methods using PRISM 3 as reported earlier and represent the mean from at least three different determinations. hCA I, hCA II, hCA IX, and hCA XII (catalytic domain) were recombinant proteins produced in-house using our standardized protocol and their concentration in the assay system was in the range of 3–10 nM. AAZ was used as reference CA inhibitor [16] [17].

## 3.2. Cellular toxicity assay

Human hepatoma-derived HuH-7 cells were maintained in Dulbecco's modified Eagle's medium (DMEM, Gibco) supplemented with 10 % v/v inactivated fetal beef serum (FBS HI, Gibco) and 1X Pen-strep (Euroclone) and kept under 5 % $CO_2$ on 37 ◦C. The cells were seeded at 200,000 cells/well in 96-well transparent cell-treated plates (Euroclone). Cells were incubated with the compound at different concentrations the following day. Compound was dissolved in 0.1 % dimethyl sulfoxide (DMSO). After 24, 48, and 72 hours post-treatment, 20 µl of 3-(4,5-dimethylthiazol-2-yl)-2,5-diphenyl-2H-tetrazolium bromide (MTT) dissolved in PBS at 7,5 mg/ml, were added to each well and the cells were incubated at 37°C with 5% $CO_2$ for 1h. Then the supernatant was removed, and cells were lysed with 100 µl/well of lysis buffer (100% 2-Propanol, 0,004% Triton-X-100, 0,0004% Hydrogen Chloride) until the formazan crystals were completely dissolved, then the absorbance was read at 570 nm with a plate reader (Victor Nivo).

Data analysis for cytotoxicity assay: The compound half-maximal cytopathic concentration ($CC_{50}$) was determined via non-linear regression using Prism 9 v. 9.4.1 software (GraphPad) with the function dose-response curve, log-concentration-normalised response.

The cytotoxic effect of the compound 3h was evaluated on Huh-7 cell line monitoring cell viability

after 24, 48 and 72 hours post-treatment (Table S5). Untreated cells were used as viability control. Camptothecin was used as a positive control for the experiment. Cells were treated with different concentrations of compound 3h for 24, 48, and 72 hours.

The results showed a weak toxicity of the compound **3h** up to 100 µM, with a residual cell viability of 60 % at 72 hours post-treatment.

**Table S5.** Investigation of the cytotoxic effect of compound **3h**.

| **Name** | **HuH-7 [a]$CC_{50}$ (µM) 24h** | **HuH-7 $CC_{50}$ (µM) 48h** | **HuH-7 $CC_{50}$ (µM) 72h** |
|---|---|---|---|
| **3h** | >100 (70%)[b] | >100 (60%) | >100 (60%) |
| Camptothecin | >10 | 8.5 ± 0.9 | 0.5 ± 0.02 |

*[a] Compound concentration required to reduce HuH-7 cells viability by 50%;*

*[b] Percentage of HuH-7 cells viability at the highest compound concentration tested.*

*Values are expressed as means ± SD of at least two independent experiments performed in triplicate.*

## 4. References


1. SAINT Version 8.37 A; Bruker AXS Inc.: Wisconsin (USA).
2. SADABS Version 2016/2; Bruker AXS Inc.: Wisconsin (USA).
3. Sheldrick, G., *SHELXT - Integrated space-group and crystal-structure determination.* Acta Crystallographica Section A, 2015. **71**(1): p. 3-8.
4. Sheldrick, G., *Crystal structure refinement with SHELXL.* Acta Crystallographica Section C, 2015. **71**(1): p. 3-8.
5. Dolomanov, O.V., et al., *OLEX2: a complete structure solution, refinement and analysis program.* Journal of Applied Crystallography, 2009. **42**(2): p. 339-341.
6. Schrödinger Release 2024-1: Protein Preparation Wizard; Epik, S., LLC, New York, NY, 2024; Impact, Schrödinger, LLC, New York, NY; Prime, Schrödinger, LLC, New York, NY, 2024. .
7. Schrödinger Release 2021-4: MacroModel; Schrödinger, L.N.Y., NY, USA, 2021.
8. Halgren, T.A., *Merck molecular force field. I. Basis, form, scope, parameterization, and performance of MMFF94.* Journal of Computational Chemistry, 1996. **17**(5-6): p. 490-519.

9. Kollman, P.A., et al., *Calculating Structures and Free Energies of Complex Molecules: Combining Molecular Mechanics and Continuum Models.* Accounts of Chemical Research, 2000. **33**(12): p. 889-897.
10. Friesner, R.A., et al., *Glide: A New Approach for Rapid, Accurate Docking and Scoring. 1. Method and Assessment of Docking Accuracy.* Journal of Medicinal Chemistry, 2004. **47**(7): p. 1739-1749.
11. Glide, S., LLC, New York, NY, USA, 2021; Prime, S., LLC, New York, NY, USA, 2021.
12. Kollman, P.A., et al., *Calculating structures and free energies of complex molecules: combining molecular mechanics and continuum models.* Acc Chem Res, 2000. **33**(12): p. 889-97.
13. Prime; Schrodinger, L.N.Y., NY, USA, 2024-1.
14. Schrödinger Release 2024-2 QikProp S, N.Y., NY, 2024.
15. Khalifah, R.G., *The carbon dioxide hydration activity of carbonic anhydrase. I. Stop-flow kinetic studies on the native human isoenzymes B and C.* J Biol Chem, 1971. **246**(8): p. 2561-73.
16. Supuran, C.T., *Carbonic anhydrases: novel therapeutic applications for inhibitors and activators.* Nat Rev Drug Discov, 2008. **7**(2): p. 168-81.
17. Meleddu, R., et al., *Selective inhibition of carbonic anhydrase IX and XII by coumarin and psoralen derivatives.* Journal of Enzyme Inhibition and Medicinal Chemistry, 2021. **36**: p. 685-692.
